# In silico mutational analysis of ACE2 to check the susceptibility of lung cancer patients towards COVID-19

**DOI:** 10.1038/s41598-022-11805-5

**Published:** 2022-05-12

**Authors:** Zumama Khalid, Abeedha Tu-Allah Khan, Radwan Alnajjar, Eman Santali, Abdul Rauf Shakoori

**Affiliations:** 1 School of Biological Sciences, University of the Punjab, Quaid-i-Azam Campus, Lahore, 54590 Pakistan; 2 Department of Chemistry, Faculty of Science, University of Benghazi, Benghazi, Libya; 3 Department of Chemistry, University of Cape Town, Rondebosch, 7701 South Africa; 4 College of Pharmacy, Taif University, P.O. Box 11099, Taif, 21944 Saudi Arabia

**Keywords:** Computational biology and bioinformatics, Immunology

## Abstract

Being the second major cause of death worldwide, lung cancer poses a significant threat to the health of patients. This worsened during the era of pandemic since lung cancer is found to be more prone to SARS-CoV-2 infection. Many recent studies imply a high frequency of COVID-19 infection associated severe outcome. However, molecular studies are still lacking in this respect. Hence the current study is designed to investigate the binding affinities of ACE2 lung cancer mutants with the viral spike protein to find the susceptibility of respective mutants carrying patients in catching the virus. Quite interestingly, our study found lesser binding affinities of all the selected mutants thus implying that these cancer patients might be less affected by the virus than others. These results are opposed to the recent studies’ propositions and open new avenues for more in-depth studies.

## Introduction

Lung cancer is the second cause of death in males and females after cardiovascular diseases worldwide^1^. Among cancers, lung cancer is the first leading cause of death among men and the second leading cause of death among women^2^. Deaths due to lung cancer in women of developed countries are higher as compared to developing countries^3^. The worldwide annual estimate of people diagnosed with lung cancer is 1.8–2.0 million, and 1.6 million people fail to survive^4^. Lung cancer is considered one of the deadliest diseases due to its delayed diagnosis, disease setback, and lack of curative medication^5^.

Lung cancer has been one of the widely malignant cancers in the entire world because of its diagnosis at advanced stages. Multiple steps are involved in the mechanism of metastasis of such wide-spreading cancer. These steps require crucial mechanisms such as angiogenesis, the transition of cells from endothelial form to mesenchymal form (EMT) by the interruption in cell to cell and cell to matrix attachment, apoptosis, and movement of cells to a secondary site. Genetic involvement in these mechanisms and pathways ensures lung cancer to be invasive and highly metastatic^6^. Lung cancer is a multifactorial disease; hence together, environmental and genetic elements play a vital role in its susceptibility^7^. Various genome-wide association studies have pointed out the multiple single nucleotide polymorphisms (SNP) in association with lung carcinoma, especially on 6p21.33, 15q25.1, and 5p15.33^8,9^.

**Figure 1:**
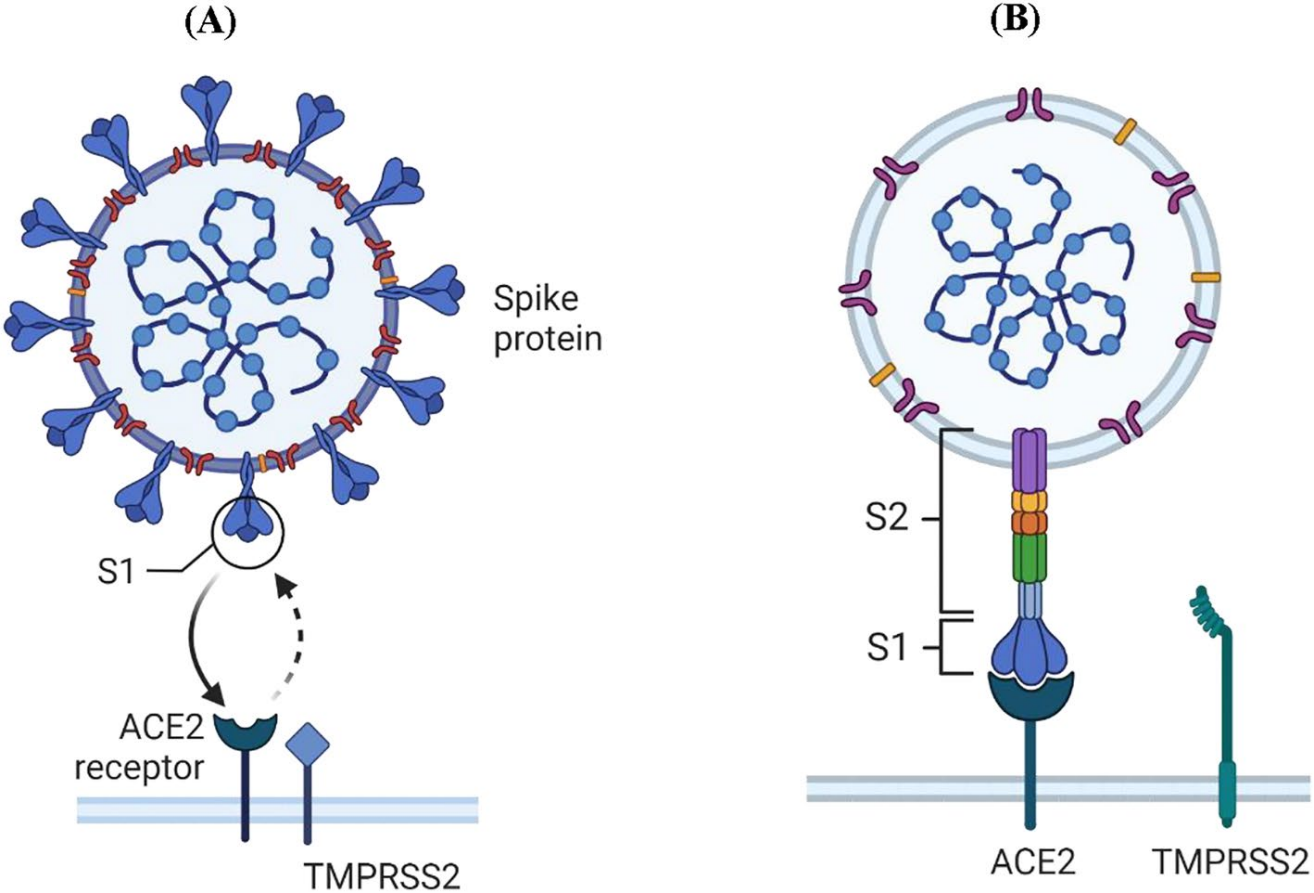
The structure of SARS-CoV-2 and the ACE2 receptor (Created with BioRender.com).

In November 2019, a novel virus emerged from Wuhan (China). This virus was labeled as Severe acute respiratory syndrome coronavirus 2(SARS-CoV-2) by the International Committee on Taxonomy of Viruses^10^. The viral infection starts from the binding of viral spike protein to the receptor site on the surface of the host. For spike protein (SARS-CoV-2 glycoprotein) of COVID-19, the identified receptor is ACE2 (Angiotensin Converting Enzyme 2) (Fig. 1A)^11^. Spike protein has two subunits S1 and S2; the S1 subunit bears the receptor-binding-domain (RBD). RBD binds directly to the peptidase domain of the ACE2 receptor. After this binding site on the S2 subunit exposes, which gets cleaved by the host protease, and viral infection is initiated (Fig. 1B)^12^. ACE2 enzyme catalyzes the conversion of angiotensin to other different forms^13,14^.

Cancer patients are seen to be more prone to COVID-19 than normal people due to their immunocompromised health status. Another reason found of being lung cancer patients more prone to COVID-19 infection is the healthcare system where contact with COVID-19 infection was more during cancer treatment^15^. There is a high frequency of severe symptoms due to COVID-19 in patients with having lung carcinoma, hematologic cancer, and metastatic cancer^11^. A high fatality rate was observed in lung cancer patients as compared to other cancer patients having COVID-19^16–19^. Although recent studies have stated that COVID-19 patients who have cancer have a high possibility of severe outcomes than patients without cancer, but there is no study pointing towards the possible molecular mechanism behind it. However, Yu et al. reported that lung cancer patients have more severity of the viral disease than other patients as the major entry route for the virus is through the lungs, and that too with reasons unknown^16–19^.

Therefore, this work has been designed to investigate the binding affinities of the viral spike protein toward mutated ACE2 proteins to decipher the susceptibility and probable molecular mechanism of the COVID-19 infection concerning ACE2.

## Materials and methods

### ACE2 mutants and SARS-CoV2 spike protein

The 3D structures of ACE2 and SARS-CoV2 spike protein were retrieved from RCSB-PDB. The RCSB-PDB code for the structures of SARS-CoV2 spike protein and ACE2 is 6M17^20^, Missense mutations and frameshift mutations in the ACE2 gene in lung tissues were curated from the COSMIC cancer database. All the mutations in the study were used in accordance with the database regulations and guidelines (https://cancer.sanger.ac.uk/cosmic). These mutations were induced in the 3D structure of ACE2 through mutagenesis in PyMOL^21^. Structures were visualized via UCSF Chimera Version 1.14^22^.

### Protein–protein docking

Protein–protein docking was performed on the HADDOCK server (https://wenmr.science.uu.nl/haddock2.4/), a web server for the docking of biomolecular structures, and the obtained binding affinities of the docked poses of the spike glycoprotein of SARS-CoV-2 with all the cancer mutants of ACE2 were analyzed^23^. Binding residues of ACE2 (24,27,28,30,31,34,35,37,38,41,42,79,82,83,330,353,354,355,357,393) were docked with binding residues of RBD (Receptor Binding Domain) of SARS-CoV2 spike protein (486,487,489,456,455,475,417,493,453,505,501,449,496,502,500,496,446) along with wild type ACE2 and spike protein^24^. The docking postures of ACE2 cancer mutants and spike glycoprotein were visualized using PyMOL and UCSF Chimera Version 1.14^21,22^.

### Analysis of docking

Based on HADDOCK scores, the top 5 docked complexes of ACE2 cancer mutants and spike protein with higher docking scores were assessed for stability and binding affinity (kcal mol^−1^). This analysis was performed on Protein Binding Energy Prediction (PRODIGY) server^25,26^. PRODIGY predicts stability and binding affinity of docking complex based on structural properties of both interacting molecules in complex. Binding affinity was demonstrated by ΔG (kcal mol^−1^) and stability by the dissociation constant Kd (M). Analysis was done at different temperature ranges. Docked complexes with higher binding affinity were subjected to molecular dynamics simulation.

### Molecular dynamic simulation and MM-GBSA

The molecular dynamic simulations were carried out using the Desmond simulation package of Schrödinger LLC^27^. Molecular dynamic simulations and MM-GBSA calculations were described in detail in the supplementary information, and it was conducted according to previous work^28–30^

### Ethical declaration

Ethical approval was not required since no animal or human subjects were used directly in the current study. Online public computational data was used in accordance with the guidelines of the COSMIC cancer database (https://cancer.sanger.ac.uk/cosmic).

## Results and discussion

### ACE2 mutants

Mutations found in ACE2 were of different types, including deletion, substitution, and insertion. The COSMIC database provided a set of mutations in the ACE2 gene in lung tissues, as depicted in Table 1; out of them, only missense and frameshift mutations were curated because they lead to change the resultant protein.

**Table 1:** Mutations for the ACE2 gene curated from the COSMIC database.

Serial no.	Position	Mutation (CDC)	Mutation (AA)	Mutation type	References
1	34	c.39T>C	p.H34N	Substitution—missense	^31^
2	47	c.140 C>G	p.S47C	Substitution—missense	^32^
3	60	c.178 C>G	p.Q60E	Substitution—missense	^31^
4	84	c.250 C>G	p.P84A	Substitution—missense	^33^
5	99	c.295 G>T	p.A99S	Substitution—missense	^31^
6	147	c.440 G>T	p.G147V	Substitution—missense	^31^
7	190	c.568 A>T	p.M190L	Substitution—missense	^32^
8	211	c.631 G>T	p.G211W	Substitution—missense	^31^
9	219	c.656 G>C	p.R219P	Substitution—missense	^31^
10	253	c.757 C>A	p.P253T	Substitution—missense	^32^
11	256	c.768 C>G	p.I256M	Substitution—missense	^31^
12	320	c.958 C>T	p.L320F	Substitution—missense	^31^
13	357	c.1071 G>T	p.R357S	Substitution—missense	^34^
14	394	c.1180 A>G	p.N394D	Substitution—missense	^35^
15	395	c.1184 G>T	p.G395V	Substitution—missense	^36^
16	398	c.1192 G>A	p.E398K	Substitution—missense	^37^
17	44	c.1330 C>T	p.L444F	Substitution—missense	^38^
18	477	c.1429 T>C	p.W477R	Substitution—missense	^36^
19	491	c.1471 G>T	p.V491L	Substitution—missense	^36^
20	670	c.2008 G>T	p.V670L	Substitution—missense	^31^
21	693	c.2077 G>A	p.D693N	Substitution—missense	^39^
22	710	c.2082 C>A	p.R710C	Substitution—missense	^40^
23	720	c.2128 C>T	p.N720D	Substitution—missense	^32^
24	768	c.2303 G>T	p.R768L	Substitution—missense	^34^
25	798	c.2392 A>C	p.T798P	Substitution—missense	^39^

### Predicted structures of SARS-CoV-2 spike protein

**Figure 2:**
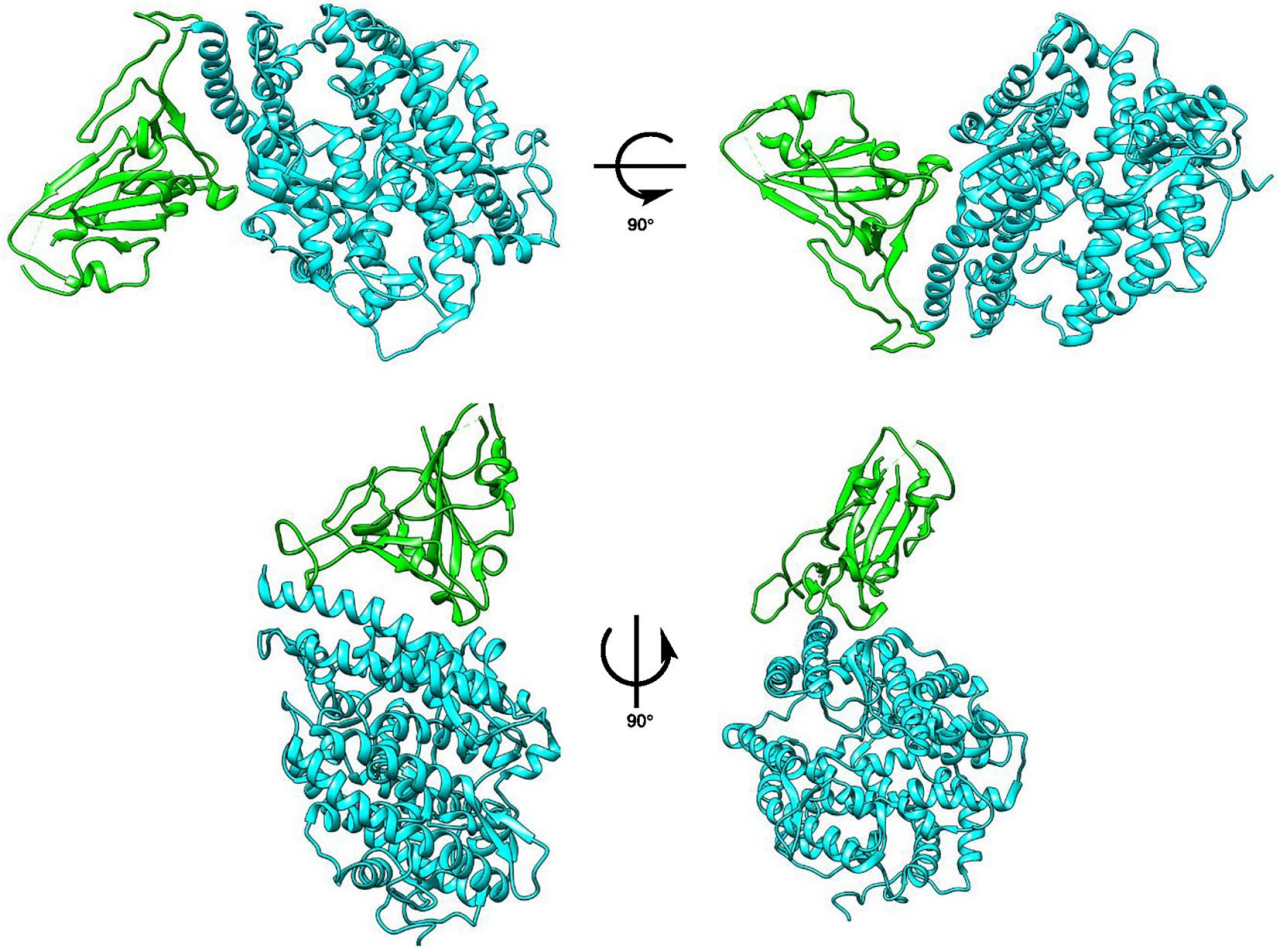
ACE2 and SARS-CoV-2 spike glycoprotein (6M17) (Cyan: ACE2; Green: RBD).

**Figure 3:**
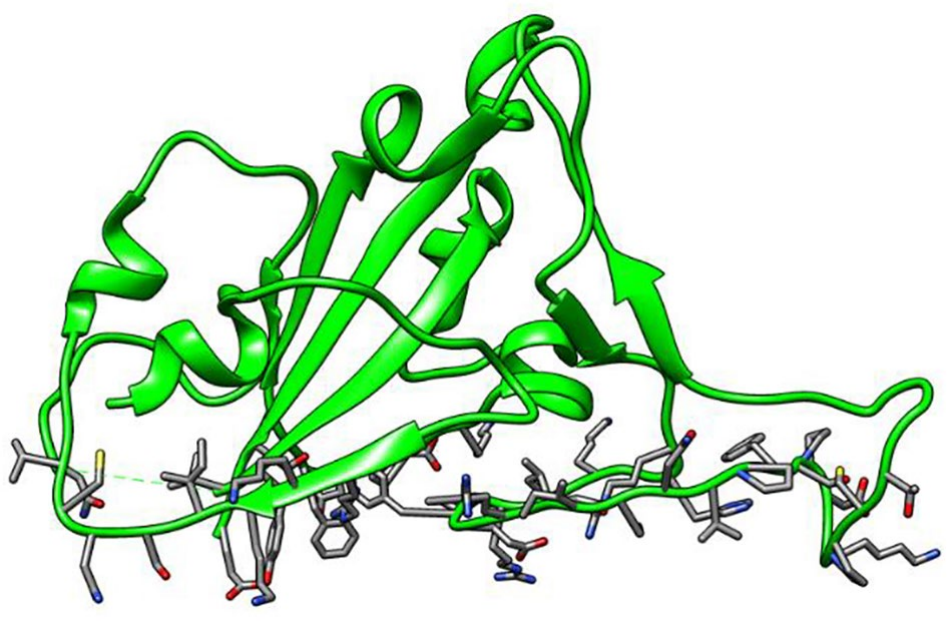
Receptor binding domain of SARS-CoV-2 spike protein.

The 3D model of SARS-CoV-2 spike glycoprotein and ACE2 were derived from RCSB PDB (6M17), Fig. 2. The spike protein contains the receptor-binding domain (RBD) in either the open or closed conformations. RBD of SARS-CoV-2 spike glycoprotein was further used for docking purposes (Fig. 3).

### Protein to protein docking

**Figure 4:**
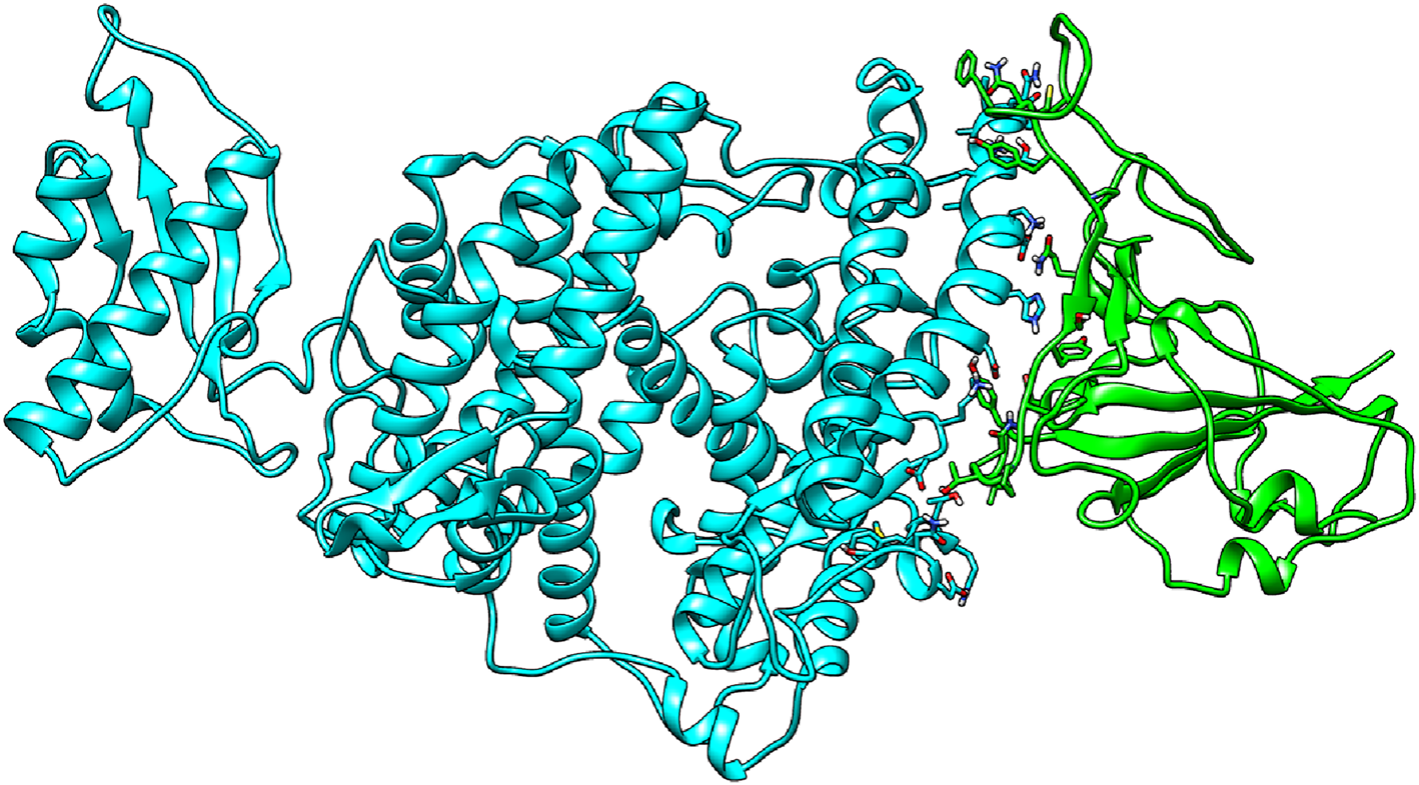
Spike–ACE2 protein interactions.

**Figure 5:**
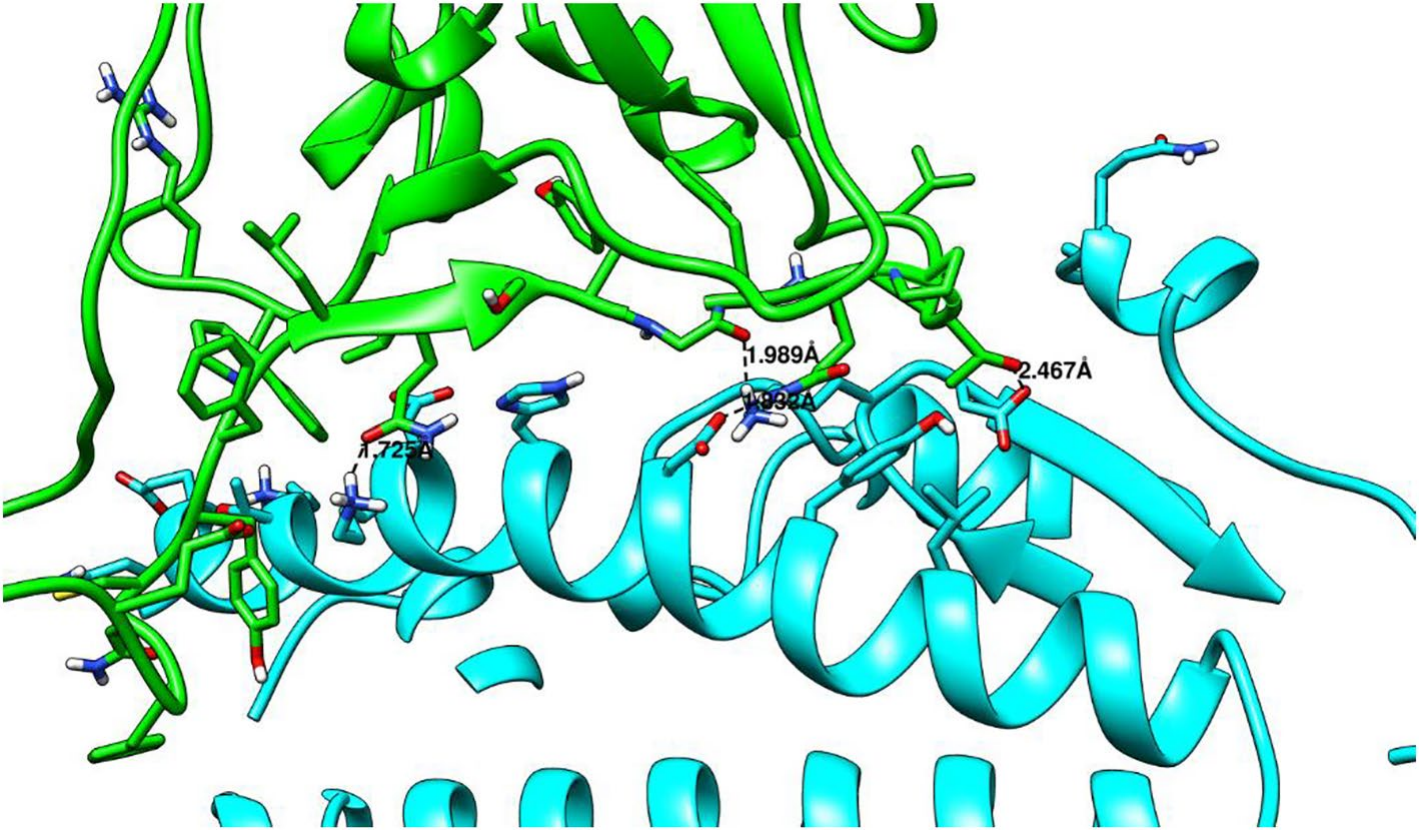
The interactions of Spike–ACE2 with highlighted hydrogen bonds.

Docking results from the HADDOCK were derived. Since minimum HADDOCK scores depict the more binding affinity of two interacting proteins, so the best poses of the docking were obtained (Figs. 4 and 5). The top five complexes out of the 5 complexes were selected based on their high HADDOCK scores than others (Table 2). Inter and intra hydrogen bonds were visualized between SARS-CoV-2 spike glycoprotein and ACE2 mutant in docking complexes.

**Table 2:** Docking results of control complex (ACE2 and SARS-CoV2 spike protein) and top 5 docked molecules (different AC2 mutants and normal SARS-CoV-2 spike protein). Docking scores are represented by HADDOCK scores (the more negative docking scores, the better the binding affinity of two proteins).

Complex	HADDOCK score	Cluster size	RMSD from the overall lowest-energy structure	Z-Score	Van der Waals energy
CNT	− 141.4 ± 6.6	38	0.9 ± 0.5	− 1.0	− 67.3 ± 5.7
P84A	− 143.7 ± 9.5	135	1.3 ± 0.7	− 1.7	− 64.9 ± 8.1
D693N	− 139.2 ± 3.2	133	0.8 ± 0.7	− 1.1	− 58.5 ± 4.5
V491L	− 138.0 ± 2.6	90	1.7 ± 1.4	− 1.3	− 61.7 ± 9.3
Q60E	− 136.8 ± 6.7	66	0.7 ± 0.6	− 1.1	− 55.9 ± 5.7
L320F	− 136.3 ± 6.6	133	1.2 ± 1.3	− 1.6	− 57.6 ± 8.1

### Protein to protein docking analysis

Due to simple scoring methods, protein docking is not reliable enough for the prediction of binding affinity between two interacting proteins in complexes^41^. The binding affinity of two proteins in a complex relies on dissociation constant (Kd), temperature and pH also^42^. These factors are not included in docking scoring methods of docking servers. Therefore, the binding affinity of frontier complexes (top 5) of interacting partners with the lowest docking scores was checked through the PRODIGY server (Tables 3, 4, 5, 6), binding affinity of all 5 complexes at core body temperature (37 ℃) is represented in Table 3. High stability and strong binding affinity between two interacting proteins in a complex are indicated by a smaller Kd value^43^. Spike protein-ACE mutants showed high stability and binding affinity as the temperature rises, and at the optimum temperature, stability and binding affinity became constant. Whereas the lowest protein stability and binding affinity were seen at 25℃, and highest on the optimum temperature (Table 3, Tables SI 3–8). Other type of interactions which may affect the binding affinity (Kd), and stability (ΔG) of docked complexes are also represented in Table 4.

**Table 3:** Binding affinity of docking complex and its dissociation constant (Kd) at 37 ℃. The binding affinity and stability of docked proteins are calculated in the form of ΔG (kcal mol^−1^) and Kd (M), respectively. Smaller Kd value is showing high stability and strong binding affinity between two proteins.

Temperature (℃)	Protein–protein complex	ΔG (kcal mol^−1^)	Kd (M)
37	CNT	− 11.5	7.8 × 10^–9^
37	P84A	− 11.8	5.0 × 10^–9^
37	D693N	− 12.0	3.6 × 10^–9^
37	V491L	− 12.9	8.3 × 10^–10^
37	L320F	− 10.9	2.1 × 10^–8^
37	Q60E	− 10.9	1.9 × 10^–8^

**Table 4:** Type of interactions affecting the binding affinity (Kd), and stability (ΔG) of docked complexes at 37 ℃.

Protein–protein complex	Number of interfacial contacts (ICs) per property						Non interacting surface (NIS) per property	
	ICs charged-charged	ICs charged-polar	ICs charged-apolar	ICs polar-polar	ICs polar-apolar	ICs apolar-apolar	NIS charged (%)	NIS apolar (%)
CNT	2	10	22	6	20	16	26.7	24.83
P84A	2	12	31	7	20	19	25.39	38.08
D693N	3	12	26	4	20	12	25.46	37.98
V491L	5	11	22	2	23	22	25.78	37.46
L320F	3	11	25	8	19	17	25.78	37.61
Q60E	3	11	26	7	18	16	26.07	37.46

**Table 5:** The average of H-bond interactions between the SARS-CoV-2 spike and ACE 2 in the last 50 ns of simulation time.

Complex	CNT	D639N	L320F	P84A	Q60E	V491L
Average H-bonds	6.1	10.6	18.9	12.8	15.6	4.7
H-bond at 500 ns	5	11	14	12	15	4

**Table 6:** Prime MM-GBSA energies for Ligands binding at the active site of COVID-19 main protease.

Complex	ΔG binding	Coulomb	Covalent	H-bond	Lipo	Packing	Solv_GB	VdW
CNT	− 87.62	− 58.59	4.64	− 5.87	− 25.38	− 2.32	101.79	− 102.82
D693N	− 69.30	− 65.86	5.37	− 6.76	− 9.70	− 1.35	99.72	− 92.29
Q60E	− 79.59	− 69.59	11.16	− 7.70	− 20.11	− 2.97	108.04	− 98.95
V491L	− 72.51	− 7.00	3.72	− 4.90	− 20.73	− 2.61	53.28	− 94.95
L320F	− 86.61	− 46.38	0.19	− 6.54	− 20.96	− 2.84	80.00	− 90.16
P84A	− 75.96	− 28.72	6.28	− 7.48	− 20.32	− 4.02	64.48	− 86.32

*Coulomb* Coulomb energy, *Covalent* Covalent binding energy, *VdW* Van der Waals energy, *Lipo* Lipophilic energy, *Solv_GB* Generalized Born electrostatic solvation energy, *H-bond* Hydrogen-bonding energy.

A change in the dissociation constant of different mutants was noted. While a decrease of 1 log fold was observed for V491L, an increase of 2 log folds was present in L320F and Q60E. However, the Kd for mutants P84A and D693N were similar to those of controls (Table 3). Moreover, a slight increase in the Kd was also observed with increasing temperatures for mutants D693N, V491L, L320F, and Q60E (Tables SI 3–8). The reason for this might be increased energy imparted with the increasing temperatures, which might be making the complexes less stable, hence an increase in Kd. Another important point to infer from these is the change in binding affinities with physiological temperatures of the human body under different states, such as normal hypothermic and hyperthermic conditions. The results also imply the less binding affinities of these ACE2 mutants under hyperthermic conditions, such as fever, and vice versa. A similar study has been performed by Basit and colleagues, where the binding affinity of ACE2 was shown to be constant at different temperatures; however, the dissociation constant was shown to be increased as the temperature increased^44^.

### Molecular dynamic simulations

Docking procedures consider rigid and lack the free movement of the protein; thus, molecular dynamics simulation was conducted on the best six Spike-ACE2 complexes in an attempt to better understanding and validation of the docking results. The MD (Molecular Dynamics) simulations were running for a period of 500 ns, and the root means square deviation RMDS for the complex, the SARS-CoV-2 Spike, and the ACE2 were reported and analyzed. The total energy along with the potential energy of each system was monitored during the simulation and the average was reported in Table S1.

### Proteins and complex Root Mean Square Deviation (RMSD) analysis

**Figure 6:**
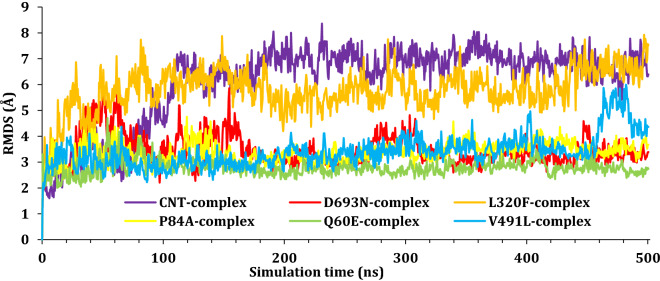
The RMSD for Cα atoms (Å) with respect to the initial structure as a function of simulation time (ns) for the six complexes.

**Figure 7:**
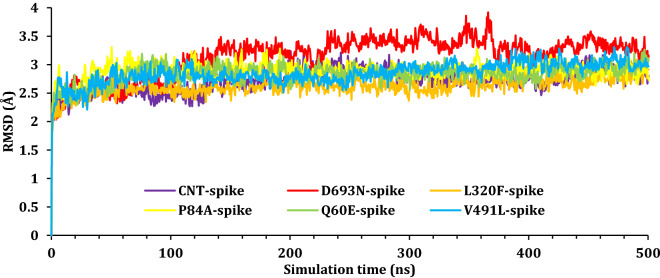
The RMSD for Cα atoms (Å) with respect to the initial structure as a function of simulation time (ns) for the SARS-CoV-2 spike protein.

**Figure 8:**
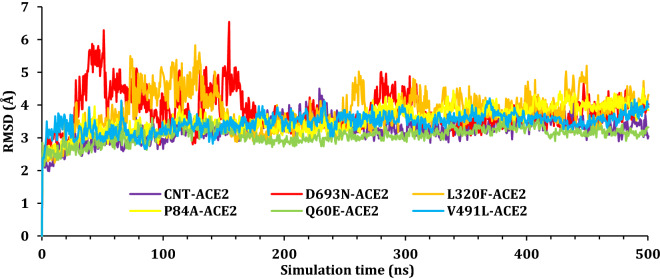
The RMSD for Cα atoms (Å) with respect to the initial structure as a function of simulation time (ns) for the ACE2 protein.

To monitor the impact of simulation on the stability of the SARS-CoV-2 Spike–ACE2 complexes, the root mean square deviations values were reported as a function of time for all C_α_ atoms of the proteins with respect to their initial positions. The RMSD results for SARS-CoV-2 Spike–ACE2 complexes were plotted as a function of simulation time and presented in Fig. 6, while the RMSD for the SARS-CoV-2 spike protein was presented in Fig. 7; finally, the RMSD of the ACE2 was plotted in Fig. 8. As it can be seen from Fig. 6, most complexes showed a stable most complexes showed stability with RMSD around 3–4 Å except for CNT-complex and L320F-complex. The CNT-complex, D693N-complex, and the L320F–complex fluctuated till around 100 ns, 180 ns, and 450 ns, respectively. Other complexes showed stability at an early stage of the simulation and held that stability during the simulations; at around 470–490 ns. A jump in the RMSD for V491L was observed and due to the movement of the ACE domain.

Further, the RMSD of each chain was studied individually with respect to its original position within the complex. Figure 7 shows the fluctuation of the spike domain of the protein–protein complex; as it can see most spike domains are at around 3 Å, which is acceptable fluctuations for proteins with such size. The only notable change occurs within the D693N Spike, and this fluctuation is due to the movement of the nonstructural part (loops) of the protein Figure SI1 (“Supplementary information”).

Figure 8 presents the RMDS of the ACE2 protein moiety; again, most proteins reached a plateau at around 200 ns, with an RMSD of ~ 3.5 Å. ACE2–D693N showed a high fluctuation at about 20–180 ns due to the instability of the tail (N-terminal) of the ACE2 during simulation, Fig. SI2 (“Supplementary information”). Figure 4 shows the position of the SARS-CoV-2 Spike with regard to ACE 2 at the beginning of the simulation (0 ns) and the end of the simulations (500 ns). In general, all ACE 2 hold position to the SARS-CoV-2 Spike; the hydrogen bonding breaking, and formation was monitored during the simulation and will be discussed later.

**Figure 9:**
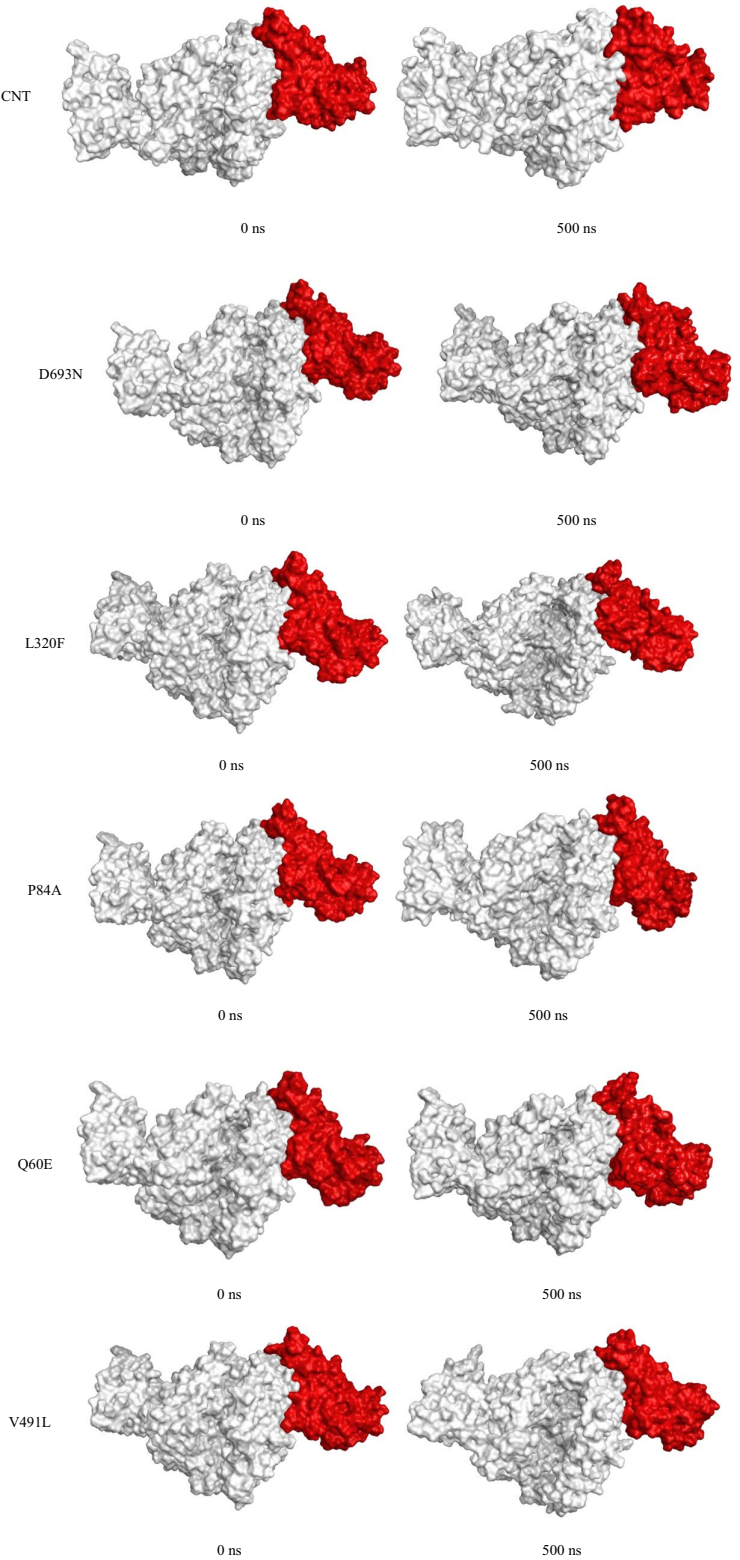
Snapshot of each complex (ACE 2, white; SARS-CoV-2 spike, red) at 0 ns and 500 ns.

Figure 9 shows a surface presentation of the first and last frame of the trajectories for a better presentation of the movement of the two proteins with respect to simulation time, and the residuals interactions will be discussed later through the course of the manuscript.

### Root mean square fluctuation (RMSF)

**Figure 10:**
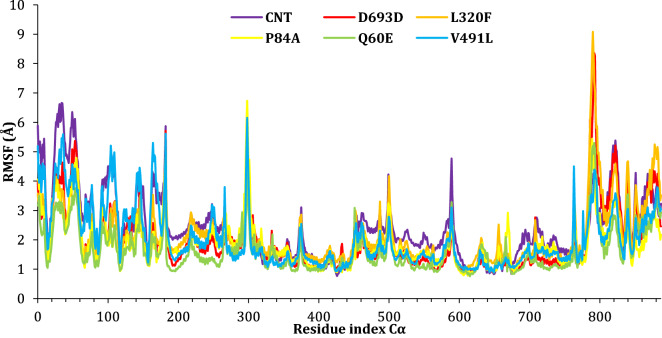
The RMSF of the complexes during simulation trajectories.

The RMSF helps characterize local changes along the protein chain. The RMSF plot peaks indicate the part of the protein that fluctuates the most during the simulation. Typically, the N- and the C- terminal fluctuates the most. Also, secondary structures like α-helices and β-strand consider more rigid than the unstructured part of the protein and fluctuate less than loops regions. Figure 10 presents the RMSF of all complexes, residue index from 0 to 182 corresponding to the SARS-CoV-2 Spike, while the residue index from 183 to 890 corresponding to ACE 2. The fluctuations between 15 and 65 corresponding to residuals ARG357 to PHE400 of the SARS-CoV-2 Spike, and the peak at 182 presents the C- terminal of the SARS-CoV-2 Spike. The peak at 298 refers to the fluctuation of ASN134, PRO135, and ASP136 of the ACE 2 protein, and the peak at 790 is due to the fluctuations of the following residuals ALA627, LEU628, and GLY629. The RMSF of L320F is provided in the supplementary information for the residual numbering example. Protein secondary structure elements (SSE) like alpha-helices and beta-strands are monitored throughout the simulation for all complexes and reported in SI 9–20.

### Hydrogen bond analysis

**Figure 11:**
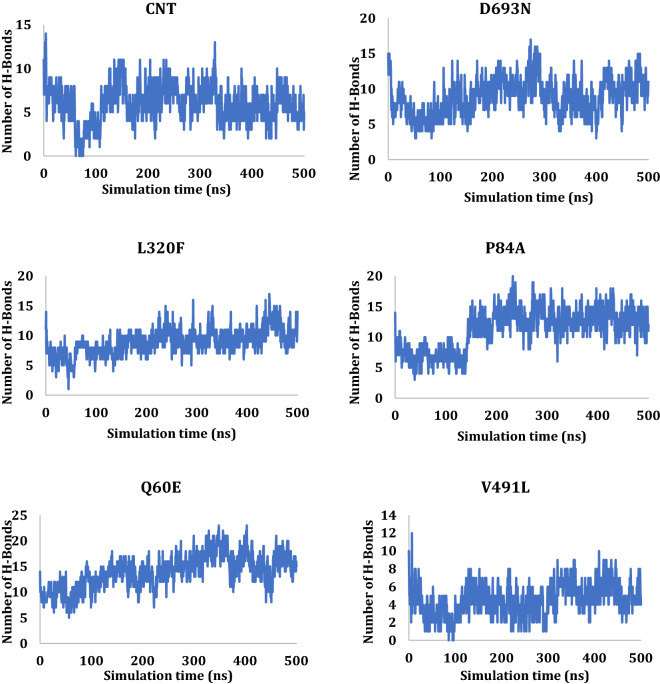
H-bonds interactions between the SARS-CoV-2 Spike and ACE 2 during simulations.

Hydrogen bonds consider the most important interactions in protein chemistry; the protein structure depends on this interaction, including 2D, 3D, and quaternary structures. The hydrogen bond between the SARS-CoV-2 Spike and the ACE 2 was monitored during the simulation time and plotted as a function of time (Fig. 8). As can be seen from Fig. 6, the H-bond between the two proteins fluctuated between 0 and 25 H-bonds, the average hydrogen bond interaction for the last 50 ns was calculated and reported in Table 5. L320F showed the most H-bond interactions with an average of 18.9 H-bonds, followed by Q60E and P84A with H-bonds interactions of 15.6 and 12.8, respectively. The number of H-bond interactions in the last frame was also reported in Table 5, and some of the residuals involved in these H-bonds were reported in Table SI 2. H-bond interactions between the SARS-CoV-2 Spike and ACE 2 during simulations, as seen in Fig. 11. Using Ligplot, all the complexes were examined for hydrogen bonding interactions between 0 and 500 ns. (Fig. 11 and SI 3–8).

### MM-GBSA calculations

Schrodinger software has a python script called thermal_mmgbsa.py, which was used to calculate the MM-GBSA from the trajectories and extract the average binding energies, including the average MM-GBSA binding energy, average Coulomb energy, average Covalent binding energy, average Van der Waals energy, average Lipophilic energy, average Generalized Born electrostatic solvation energy, and average Hydrogen-bonding energy. All the obtained energies are shown in Table 6.

As it can be seen from Table 6, CNT and L320F showed the highest binding energy with − 87.62 and − 86.61 kcal mol^−1^, respectively, while D693N showed the lowest binding energy − 69.30 kcal mol^−1^. Coulomb energy is associated with electrostatic forces of the system and reflects the ionic interactions of the system. CNT, D693N, and Q60E showed good ionic interactions, with Q60E having the highest value, while V491L seem to lose ionic interactions. Most complexes showed good H-bond energies, as well as good Van der Waals energies.

## Conclusion

In this research, the relationship between lung cancer and COVID-19 was addressed at a molecular level through computational study. The binding affinity of the viral spike protein of the SARS-CoV-2 glycoprotein towards a mutate ACE2 was investigated, using both docking and molecular dynamic simulation approaches. Among 25 selected mutations, it was found that five mutations have higher binding energies than others from a docking perspective. These five complexes’ stability was studied and investigated further considering molecular dynamics simulations. Finally, the binding free energy calculations using the MM-GBSA approach were implemented and showed that these mutations have a binding energy of the following order CNT > L320F > Q60E > P84A > V491L > D693N. These findings suggest that some cancer patients will be less affected than others, even though most reported mutations were not within the active site of interactions; the binding energy was affected by these mutations. These results somehow oppose those clinical studies which state that lung cancer patients are more prone to COVID-19 infection based on the healthcare scenario they have observed. That might be due to the contact of healthcare system with COVID-19 to lung cancer patients during treatment. However, more in-depth studies are needed to be performed to reach at a valid endpoint.

## Supplementary Information

Supplementary Information.

## Data Availability

COSMIC Cancer Database (https://cancer.sanger.ac.uk/cosmic) was used to curate ACE2 lung cancer mutations. These mutations were publicly available on the website of database to be used for research work. These mutations were further analyzed to check their susceptibility for COVID-19. Throughout the manuscript used mutations were cited properly (Table 1). According to the rules and regulations of the database, data can be used for research purposes and correct citation is required, and so it was the done the same.
