# Supplementary material for: In silico mutational analysis of ACE2 to check the susceptibility of lung cancer patients towards COVID-19
Source: Sci Rep. 2022 May 12;12:7798. doi: 10.1038/s41598-022-11805-5 (PMC9098448; doi:10.1038/s41598-022-11805-5)
Supplement: Supplementary file 1 — Supplementary Information. [file 41598_2022_11805_MOESM1_ESM.docx]

*In silico* mutational analysis of ACE2 to check the susceptibility of lung cancer patients towards COVID-19

**Zumama Khalid^1^, Abeedha Tu-Allah Khan^1^*, Radwan Alnajjar^2,3^, Eman Santali^4^ & Abdul Rauf Shakoori^1^**

1 - School of Biological Sciences, University of the Punjab, Quaid-i-Azam Campus, Lahore 54590, Pakistan.

2 - Department of Chemistry, Faculty of Science, University of Benghazi, Benghazi, Libya.

3 - Department of Chemistry, University of Cape Town, Rondebosch 7701, South Africa.

4 - College of Pharmacy, Taif University, P.O. Box 11099, Taif 21944, Saudi Arabia.

**List of Tables**

[Table SI 1: The molecular dynamics simulations energies of each system 3](#_Toc80404008)

[Table SI 2: Some residuals that involved in the H-bond interactions at 500ns 4](#_Toc80404009)

[Table SI 3: Binding affinity of P84A and its dissociation constant (Kd) at different temperatures. The binding affinity and stability of docked proteins are calculated in the form of ΔG (kcal mol^-1^) and Kd (M), respectively. A smaller Kd value is showing high stability and strong binding affinity between two proteins. 5](#_Toc80404010)

[Table SI 4: Binding affinity of D693N and its dissociation constant (Kd) at different temperatures. The binding affinity and stability of docked proteins are calculated in the form of ΔG (kcal mol^-1^) and Kd (M), respectively. A smaller Kd value is showing high stability and strong binding affinity between two proteins. 5](#_Toc80404011)

[Table SI 5: Binding affinity of V491L and its dissociation constant (Kd) at different temperatures. The binding affinity and stability of docked proteins are calculated in the form of ΔG (kcal mol^-1^) and Kd (M), respectively. A smaller Kd value is showing high stability and strong binding affinity between two proteins. 6](#_Toc80404012)

[Table SI 6: Binding affinity of L320F and its dissociation constant (Kd) at different temperatures. The binding affinity and stability of docked proteins are calculated in the form of ΔG (kcal mol^-1^) and Kd (M), respectively. A smaller Kd value is showing high stability and strong binding affinity between two proteins. 6](#_Toc80404013)

[Table SI 7: Binding affinity of Q60E and its dissociation constant (Kd) at different temperatures. The binding affinity and stability of docked proteins are calculated in the form of ΔG (kcal mol^-1^) and Kd (M), respectively. A smaller Kd value is showing high stability and strong binding affinity between two proteins. 7](#_Toc80404014)

[Table SI 8: Binding affinity of control complex of ACE2 and SARS-CoV2 spike protein and its dissociation constant (Kd) at different temperatures. The binding affinity and stability of docked proteins are calculated in the form of ΔG (kcal mol^-1^) and Kd (M), respectively. A smaller Kd value is showing high stability and strong binding affinity between two proteins. 8](#_Toc80404015)

**List of Figures**

[Figure SI 1: Overlay of frame 2 (0.5 ns, red) on frame 734 (366.5 ns, green) of the spike protein in the D639N complex. 4](#_Toc83287160)

[Figure SI 2: The fluctuation of the ACE 2 tail during the simulation 4](#_Toc83287161)

[Figure SI 3: Hydrogen Bonding Analysis of Control Complex (a): 0 ns, (b): 250 ns, (c) 500 ns 10](#_Toc83287162)

[Figure SI 4: Hydrogen Bonding Analysis of D693N (a): 0 ns, (b): 250 ns, (c) 500 ns 11](#_Toc83287163)

[Figure SI 5: Hydrogen Bonding Analysis of L320F (a): 0 ns, (b): 250 ns, (c) 500 ns 13](#_Toc83287164)

[Figure SI 6: Hydrogen Bonding Analysis of P84A (a): 0 ns, (b): 250 ns, (c) 500 ns 14](#_Toc83287165)

[Figure SI 7: Hydrogen Bonding Analysis of Q60E (a): 0 ns, (b): 250 ns, (c) 500 ns 16](#_Toc83287166)

[Figure SI 8: Hydrogen Bonding Analysis of Q60E (a): 0 ns, (b): 250 ns, (c) 500 ns 17](#_Toc83287167)

[Figure SI 9: The SSE distribution by residue index throughout the protein structure for CNT 17](#_Toc83287168)

[Figure SI 10: The SSE composition for each trajectory frame over the course of the simulation, and the plot at the bottom monitors each residue and its SSE assignment over time for CNT complex. 18](#_Toc83287169)

[Figure SI 11: The SSE distribution by residue index throughout the protein structure for D693N 18](#_Toc83287170)

[Figure SI 12: The SSE composition for each trajectory frame over the course of the simulation, and the plot at the bottom monitors each residue and its SSE assignment over time for D693N complex. 19](#_Toc83287171)

[Figure SI 13: The SSE distribution by residue index throughout the protein structure for L320F 19](#_Toc83287172)

[Figure SI 14: The SSE composition for each trajectory frame over the course of the simulation, and the plot at the bottom monitors each residue and its SSE assignment over time for L320F complex. 20](#_Toc83287173)

[Figure SI 15: The SSE distribution by residue index throughout the protein structure for P84A 20](#_Toc83287174)

[Figure SI 16: The SSE composition for each trajectory frame over the course of the simulation, and the plot at the bottom monitors each residue and its SSE assignment over time for P84A complex. 21](#_Toc83287175)

[Figure SI 17: The SSE distribution by residue index throughout the protein structure for Q60E 21](#_Toc83287176)

[Figure SI 18: The SSE composition for each trajectory frame over the course of the simulation, and the plot at the bottom monitors each residue and its SSE assignment over time for Q60E complex. 22](#_Toc83287177)

[Figure SI 19: The SSE distribution by residue index throughout the protein structure for V491L 22](#_Toc83287178)

[Figure SI 20: The SSE composition for each trajectory frame over the course of the simulation, and the plot at the bottom monitors each residue and its SSE assignment over time for V491L complex. 23](#_Toc83287179)

**Materials and Methods**

**Molecular Dynamics simulations**

The MD simulations were carried out using Desmond simulation package of Schrödinger LLC.[1] The NPT ensemble with the temperature 300 K and a pressure 1 bar was applied in all runs. The simulation length was 500 ns with a relaxation time 1 ps. The OPLS3 force field parameters were used in all simulations.[2] The cutoff radius in Coulomb interactions was 20.00Å. The orthorhombic periodic box boundaries were set 10 Å away from the protein atoms., The water molecules were explicitly described using the transferable intermolecular potential with three points (TIP3P) model. [3-4] salt concentration set to 0.15 M NaCl and was built using the System Builder utility of Desmond.[5] The Martyna−Tuckerman−Klein chain coupling scheme with a coupling constant of 2.0 ps was used for the pressure control and the Nosé−Hoover chain coupling scheme for the temperature control. [6-7] Nonbonded forces were calculated using a RESPA integrator where the shortrange forces were updated every step and the long-range forces were updated every three steps. The trajectories were saved at 20 ns intervals for analysis. The behavior and interactions between the ligands and protein were analyzed using the Simulation Interaction Diagram tool implemented in the Desmond MD package. The stability of MD simulations was monitored by looking at the RMSD of the ligand and protein atom positions in time.

**MD trajectory analysis and prime MM-GBSA calculations**

simulation interactions diagram panel of Maestro software was used to monitoring interactions contribution in the ligand-protein stability. The Molecular Mechanics Generalized Born Solvent Accessibility (MM – GBSA) was performed to calculate the protein-protein binding free energies over the last 50 ns period. Thermal_mmgbsa.py python script provided by Schrodinger was used for this purpose. Which takes a Desmond trajectory file, splits it into individual snapshots, runs the MM-GBSA calculations on each frame, and outputs the average computed binding energy.

The binding energy is calculated according to the equation:

$${\Delta G}_{\mathrm{bind}}=E_{\left( minimized complex \right)}-E_{\left( minimized ligand \right)}-E_{(minimized receptor)}$$

**Results and Discussion**

Table SI 1: The molecular dynamics simulations energies of each system

| Property – average | CNT | D693N | Q60E | V491L | P84A |
| --- | --- | --- | --- | --- | --- |
| Total energy  (kcal mol^-1^) | -309694.837 | -305762.486 | -303984.228 | -310236.627 | -310236.627 |
| Potential energy (kcal mol^-1^) | -380090.557 | -375339.32 | -373105.970 | -380742.538 | -380742.538 |


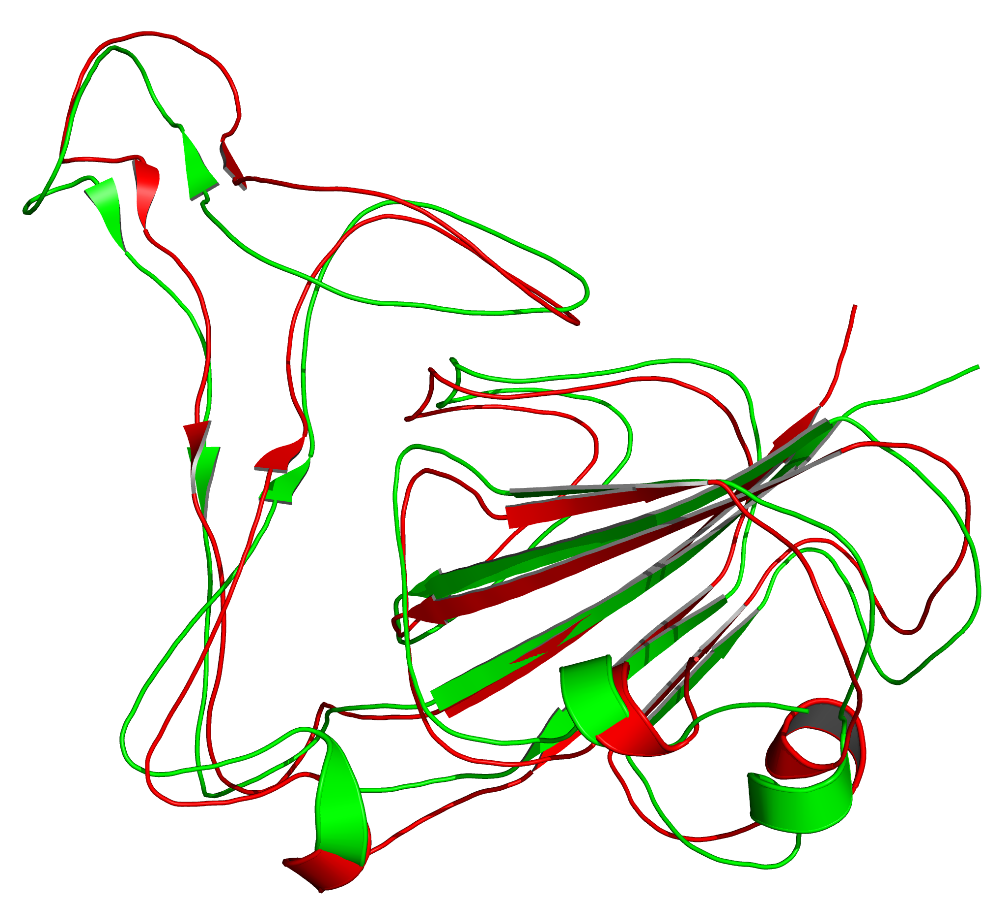


Figure SI 1: Overlay of frame 2 (0.5 ns, red) on frame 734 (366.5 ns, green) of the spike protein in the D639N complex.


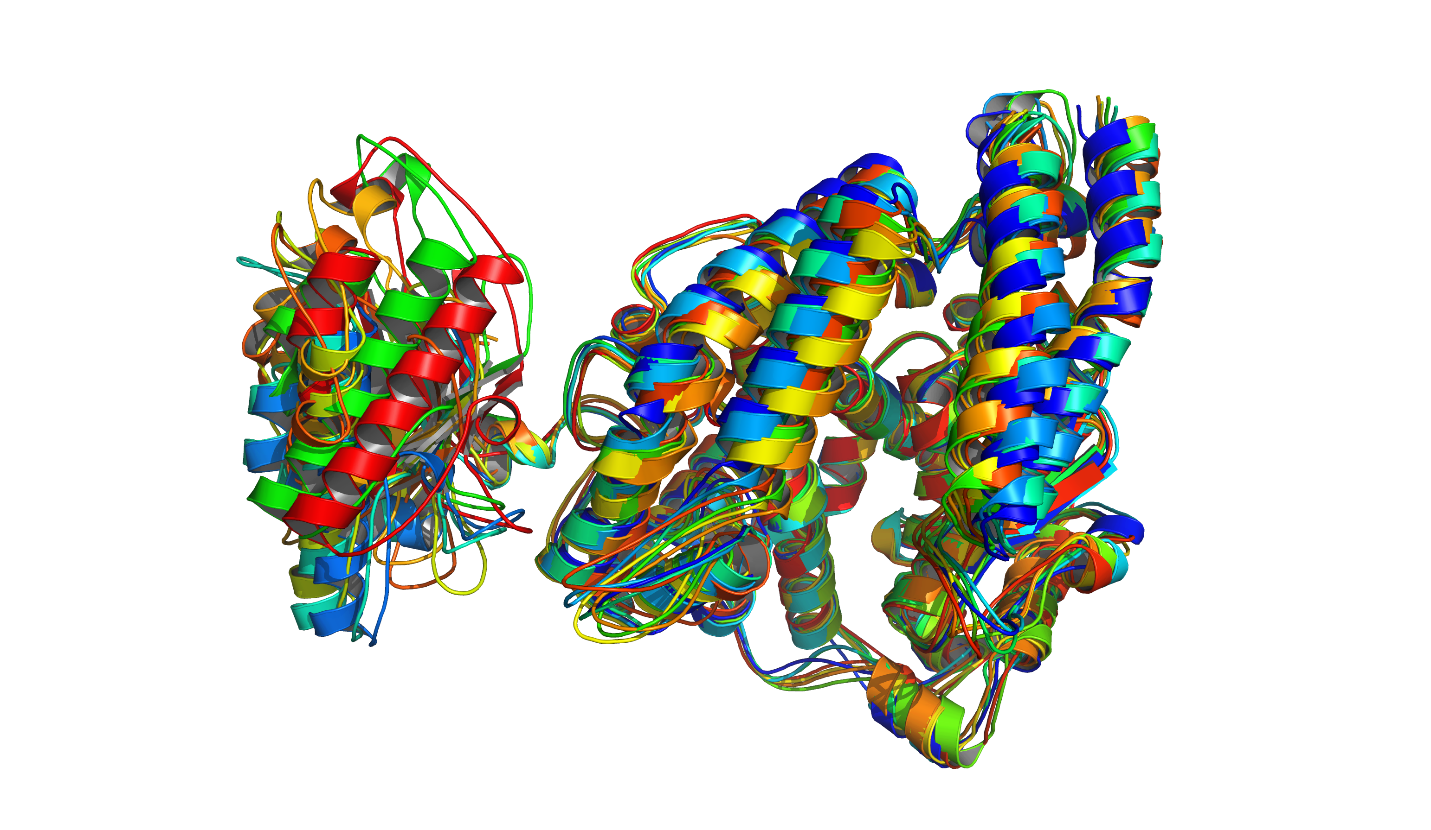


Figure SI 2: The fluctuation of the ACE 2 tail during the simulation

Table SI 2: Some residuals that involved in the H-bond interactions at 500ns

| **CNT** | | **D693N** | | **L320F** | | **P84A** | | **Q60E** | | **V491L** | |
| --- | --- | --- | --- | --- | --- | --- | --- | --- | --- | --- | --- |
| **Spike** | **ACE 2** | **Spike** | **ACE 2** | **Spike** | **ACE 2** | **Spike** | **ACE 2** | **Spike** | **ACE 2** | **Spike** | **ACE 2** |
| GLN493 | HIS34 | SER477 | GLN24 | ASN487 | TYR83 | ASN487 | TYR83 | LYS458 | GLU23 | TRY489 | TYR83 |
| TYR505 | GLN388 | THR478 | GLN24 | ASN487 | GLN24 | TYR489 | TYR83 | TYR489 | TYR83 | ASN487 | TYR83 |
| TYR505 | ARG559 | PHE486 | TYR83 | ALA475 | GLU23 | LYS458 | GLU23 | GLN493 | GLU35 | ALA475 | GLN24 |
| GLY502 | ALA384 | TYR453 | HIS34 | PHE490 | LYS31 | LYS417 | ASP30 | TYR505 | GLU37 | LYS417 | ASP30 |
|  |  | TYR505 | GLU37 | LEU492 | LYS31 | TYR453 | HIS34 | GLY502 | LYS353 |  |  |
|  |  |  |  | GLN493 | ASP38 | SER494 | HIS34 | GLN498 | ASP38 |  |  |
|  |  |  |  | ARG403 | ASP38 | GLY502 | LYS353 |  |  |  |  |
|  |  |  |  | TRY505 | GLN42 |  |  |  |  |  |  |
|  |  |  |  | ASN501 | GLN42 |  |  |  |  |  |  |
|  |  |  |  | TYR449 | LYS68 |  |  |  |  |  |  |
|  |  |  |  | GLN498 | ASN64 |  |  |  |  |  |  |

Table SI 3: Binding affinity of P84A and its dissociation constant (Kd) at different temperatures. The binding affinity and stability of docked proteins are calculated in the form of ΔG (kcal mol^-1^) and Kd (M), respectively. A smaller Kd value is showing high stability and strong binding affinity between two proteins.

| Temperature (℃) | Protein-Protein Complex | ΔG (kcal mol^-1^) | Kd (M) |
| --- | --- | --- | --- |
| 25 | P84A | -11.8 | 2.3E-09 |
| 26 | P84A | -11.8 | 2.5E-09 |
| 27 | P84A | -11.8 | 2.6E-09 |
| 28 | P84A | -11.8 | 2.8E-09 |
| 29 | P84A | -11.8 | 3.0E-09 |
| 30 | P84A | -11.8 | 3.2E-09 |
| 31 | P84A | -11.8 | 3.4E-09 |
| 32 | P84A | -11.8 | 3.6E-09 |
| 33 | P84A | -11.8 | 3.9E-09 |
| 34 | P84A | -11.8 | 4.1E-09 |
| 35 | P84A | -11.8 | 4.4E-09 |
| 36 | P84A | -11.8 | 4.7E-09 |
| 37 | P84A | -11.8 | 5.0E-09 |
| 38 | P84A | -11.8 | 5.3E-09 |
| 39 | P84A | -11.8 | 5.6E-09 |
| 40 | P84A | -11.8 | 5.9E-09 |
| 41 | P84A | -11.8 | 6.3E-09 |
| 42 | P84A | -11.8 | 6.7E-09 |
| 43 | P84A | -11.8 | 7.1E-09 |
| 44 | P84A | -11.8 | 7.6E-09 |
| 45 | P84A | -11.8 | 8.0E-09 |
| 46 | P84A | -11.8 | 8.5E-09 |
| 47 | P84A | -11.8 | 9.0E-09 |
| 48 | P84A | -11.8 | 9.5E-09 |
| 49 | P84A | -11.8 | 1.0E-09 |

Table SI 4: Binding affinity of D693N and its dissociation constant (Kd) at different temperatures. The binding affinity and stability of docked proteins are calculated in the form of ΔG (kcal mol^-1^) and Kd (M), respectively. A smaller Kd value is showing high stability and strong binding affinity between two proteins.

| Temperature (℃) | Protein-Protein Complex | ΔG (kcal mol-1) | Kd (M) |
| --- | --- | --- | --- |
| 25 | D693N | -12.0 | 1.7E-09 |
| 26 | D693N | -12.0 | 1.8E-09 |
| 27 | D693N | -12.0 | 1.9E-09 |
| 28 | D693N | -12.0 | 2.0E-09 |
| 29 | D693N | -12.0 | 2.2E-09 |
| 30 | D693N | -12.0 | 2.3E-09 |
| 31 | D693N | -12.0 | 2.5E-09 |
| 32 | D693N | -12.0 | 2.6E-09 |
| 33 | D693N | -12.0 | 2.8E-09 |
| 34 | D693N | -12.0 | 3.0E-09 |
| 35 | D693N | -12.0 | 3.2E-09 |
| 36 | D693N | -12.0 | 3.4E-09 |
| 37 | D693N | -12.0 | 3.6E-09 |
| 38 | D693N | -12.0 | 3.9E-09 |
| 39 | D693N | -12.0 | 4.1E-09 |
| 40 | D693N | -12.0 | 4.4E-09 |
| 41 | D693N | -12.0 | 4.7E-09 |
| 42 | D693N | -12.0 | 5.0E-09 |
| 43 | D693N | -12.0 | 5.3E-09 |
| 44 | D693N | -12.0 | 5.6E-09 |
| 45 | D693N | -12.0 | 5.9E-09 |
| 46 | D693N | -12.0 | 6.3E-09 |
| 47 | D693N | -12.0 | 6.7E-09 |
| 48 | D693N | -12.0 | 7.1E-09 |
| 49 | D693N | -12.0 | 7.5E-09 |
| 50 | D693N | -12.0 | 7.9E-09 |
| 51 | D693N | -12.0 | 8.4E-09 |
| 52 | D693N | -12.0 | 8.9E-09 |
| 53 | D693N | -12.0 | 9.4E-09 |
| 54 | D693N | -12.0 | 1.0E-08 |

Table SI 5: Binding affinity of V491L and its dissociation constant (Kd) at different temperatures. The binding affinity and stability of docked proteins are calculated in the form of ΔG (kcal mol^-1^) and Kd (M), respectively. A smaller Kd value is showing high stability and strong binding affinity between two proteins.

| Temperature (℃) | Protein-Protein Complex | ΔG (kcal mol-1) | Kd (M) |
| --- | --- | --- | --- |
| 25 | V491L | -12.9 | 3.6E-10 |
| 26 | V491L | -12.9 | 3.8E-10 |
| 27 | V491L | -12.9 | 4.1E-10 |
| 28 | V491L | -12.9 | 4.4E-10 |
| 29 | V491L | -12.9 | 4.8E-10 |
| 30 | V491L | -12.9 | 5.1E-10 |
| 31 | V491L | -12.9 | 5.5E-10 |
| 32 | V491L | -12.9 | 5.9E-10 |
| 33 | V491L | -12.9 | 6.3E-10 |
| 34 | V491L | -12.9 | 6.7E-10 |
| 35 | V491L | -12.9 | 9.8E-10 |
| 36 | V491L | -12.9 | 7.7E-10 |
| 37 | V491L | -12.9 | 8.3E-10 |

Table SI 6: Binding affinity of L320F and its dissociation constant (Kd) at different temperatures. The binding affinity and stability of docked proteins are calculated in the form of ΔG (kcal mol^-1^) and Kd (M), respectively. A smaller Kd value is showing high stability and strong binding affinity between two proteins.

| Temperature (℃) | Protein-Protein Complex | ΔG (kcal mol-1) | Kd (M) |
| --- | --- | --- | --- |
| 25 | L320F | -10.9 | 1.0E-08 |
| 26 | L320F | -10.9 | 1.1E-08 |
| 27 | L320F | -10.9 | 1.2E-08 |
| 28 | L320F | -10.9 | 1.2E-08 |
| 29 | L320F | -10.9 | 1.3E-08 |
| 30 | L320F | -10.9 | 1.4E-08 |
| 31 | L320F | -10.9 | 1.5E-08 |
| 32 | L320F | -10.9 | 1.6E-08 |
| 33 | L320F | -10.9 | 1.7E-08 |
| 34 | L320F | -10.9 | 1.8E-08 |
| 35 | L320F | -10.9 | 1.9E-08 |
| 36 | L320F | -10.9 | 2.0E-08 |
| 37 | L320F | -10.9 | 2.1E-08 |
| 38 | L320F | -10.9 | 2.2E-08 |
| 39 | L320F | -10.9 | 2.4E-08 |
| 40 | L320F | -10.9 | 2.5E-08 |
| 41 | L320F | -10.9 | 2.6E-08 |
| 42 | L320F | -10.9 | 2.8E-08 |
| 43 | L320F | -10.9 | 3.0E-08 |
| 44 | L320F | -10.9 | 3.1E-08 |
| 45 | L320F | -10.9 | 3.3E-08 |
| 46 | L320F | -10.9 | 3.5E-08 |
| 47 | L320F | -10.9 | 3.7E-08 |
| 48 | L320F | -10.9 | 3.9E-08 |
| 49 | L320F | -10.9 | 4.1E-08 |
| 50 | L320F | -10.9 | 4.3E-08 |
| 51 | L320F | -10.9 | 4.5E-08 |
| 52 | L320F | -10.9 | 4.8E-08 |
| 53 | L320F | -10.9 | 5.0E-08 |
| 54 | L320F | -10.9 | 5.3E-08 |
| 55 | L320F | -10.9 | 5.6E-08 |
| 56 | L320F | -10.9 | 5.9E-08 |
| 57 | L320F | -10.9 | 6.2E-08 |
| 58 | L320F | -10.9 | 6.5E-08 |
| 59 | L320F | -10.9 | 6.8E-08 |
| 60 | L320F | -10.9 | 7.2E-08 |
| 61 | L320F | -10.9 | 7.5E-08 |
| 62 | L320F | -10.9 | 7.9E-08 |
| 63 | L320F | -10.9 | 8.3E-08 |
| 64 | L320F | -10.9 | 8.7E-08 |
| 65 | L320F | -10.9 | 9.1E-08 |
| 66 | L320F | -10.9 | 9.6E-08 |
| 67 | L320F | -10.9 | 1.0E-07 |

Table SI 7: Binding affinity of Q60E and its dissociation constant (Kd) at different temperatures. The binding affinity and stability of docked proteins are calculated in the form of ΔG (kcal mol^-1^) and Kd (M), respectively. A smaller Kd value is showing high stability and strong binding affinity between two proteins.

| Temperature (℃) | Protein-Protein Complex | ΔG (kcal mol-1) | Kd (M) |
| --- | --- | --- | --- |
| 25 | Q60E | -10.9 | 9.4E-09 |
| 26 | Q60E | -10.9 | 1.0E-08 |
| 27 | Q60E | -10.9 | 1.1E-08 |
| 28 | Q60E | -10.9 | 1.1E-08 |
| 29 | Q60E | -10.9 | 1.2E-08 |
| 30 | Q60E | -10.9 | 1.3E-08 |
| 31 | Q60E | -10.9 | 1.4E-08 |
| 32 | Q60E | -10.9 | 1.4E-08 |
| 33 | Q60E | -10.9 | 1.5E-08 |
| 34 | Q60E | -10.9 | 1.6E-08 |
| 35 | Q60E | -10.9 | 1.7E-08 |
| 36 | Q60E | -10.9 | 1.8E-08 |
| 37 | Q60E | -10.9 | 1.9E-08 |

Table SI 8: Binding affinity of control complex of ACE2 and SARS-CoV2 spike protein and its dissociation constant (Kd) at different temperatures. The binding affinity and stability of docked proteins are calculated in the form of ΔG (kcal mol^-1^) and Kd (M), respectively. A smaller Kd value is showing high stability and strong binding affinity between two proteins.

| Temperature (℃) | Protein-Protein Complex | ΔG (kcal mol-1) | Kd (M) |
| --- | --- | --- | --- |
| 25 | CNT | -11.5 | 3.7E-09 |
| 26 | CNT | -11.5 | 3.9E-09 |
| 27 | CNT | -11.5 | 4.2E-09 |
| 28 | CNT | -11.5 | 4.5E-09 |
| 29 | CNT | -11.5 | 4.8E-09 |
| 30 | CNT | -11.5 | 5.1E-09 |
| 31 | CNT | -11.5 | \| 5.4E-09 \| \| --- \| |
| 32 | CNT | -11.5 | 5.8E-09 |
| 33 | CNT | -11.5 | 6.1E-09 |
| 34 | CNT | -11.5 | 6.5E-09 |
| 35 | CNT | -11.5 | 7.0E-09 |
| 36 | CNT | -11.5 | 7.4E-09 |
| 37 | CNT | -11.5 | 7.8E-09 |
| 38 | CNT | -11.5 | 8.3E-09 |
| 39 | CNT | -11.5 | 8.8E-09 |
| 40 | CNT | -11.5 | 9.4E-09 |
| 41 | CNT | -11.5 | 9.9E-09 |
| 42 | CNT | -11.5 | 1.1E-08 |

(a)

(b)

(c)

Figure SI 3: Hydrogen Bonding Analysis of Control Complex (a): 0 ns, (b): 250 ns, (c) 500 ns

(a)

(b)

(c)

Figure SI 4: Hydrogen Bonding Analysis of D693N (a): 0 ns, (b): 250 ns, (c) 500 ns

(a)

(b)

(c)

Figure SI 5: Hydrogen Bonding Analysis of L320F (a): 0 ns, (b): 250 ns, (c) 500 ns

(a)

(b)

(c)

Figure SI 6: Hydrogen Bonding Analysis of P84A (a): 0 ns, (b): 250 ns, (c) 500 ns

(a)

(b)

(c)

Figure SI 7: Hydrogen Bonding Analysis of Q60E (a): 0 ns, (b): 250 ns, (c) 500 ns

(a)

(b)

(c)

Figure SI 8: Hydrogen Bonding Analysis of Q60E (a): 0 ns, (b): 250 ns, (c) 500 ns


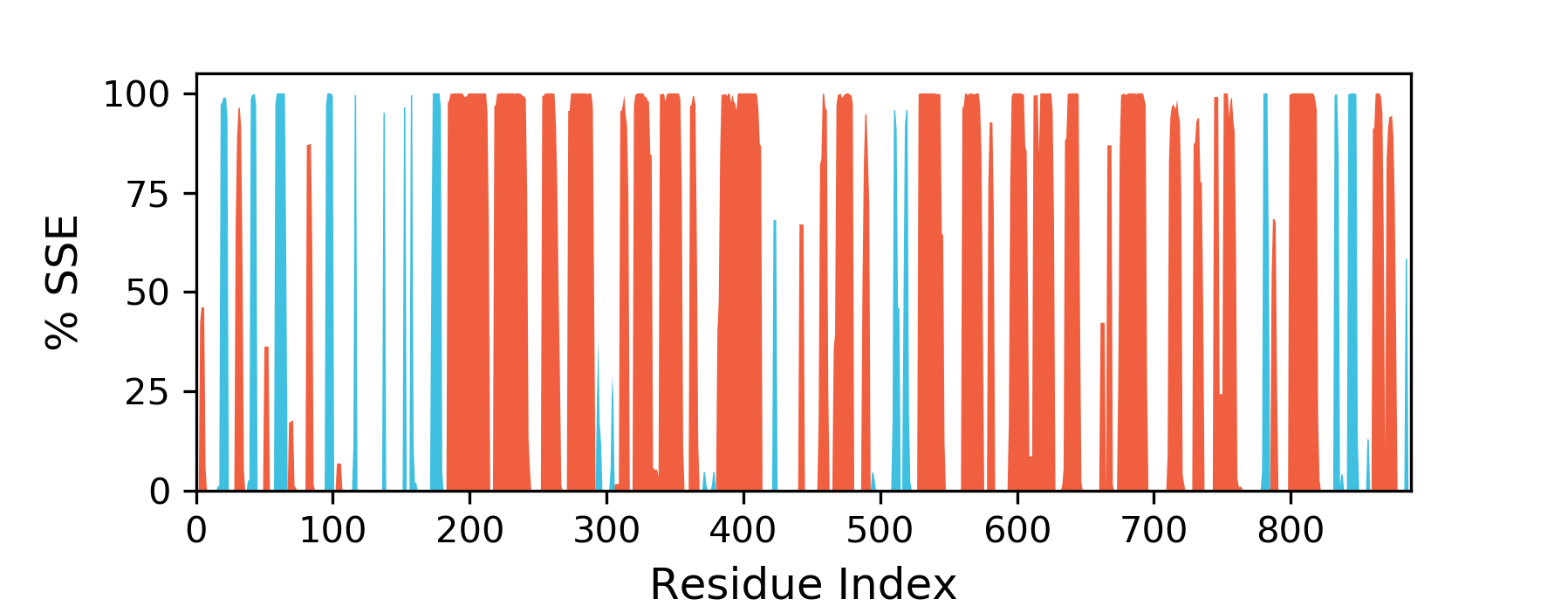


Figure SI 9: The SSE distribution by residue index throughout the protein structure for CNT


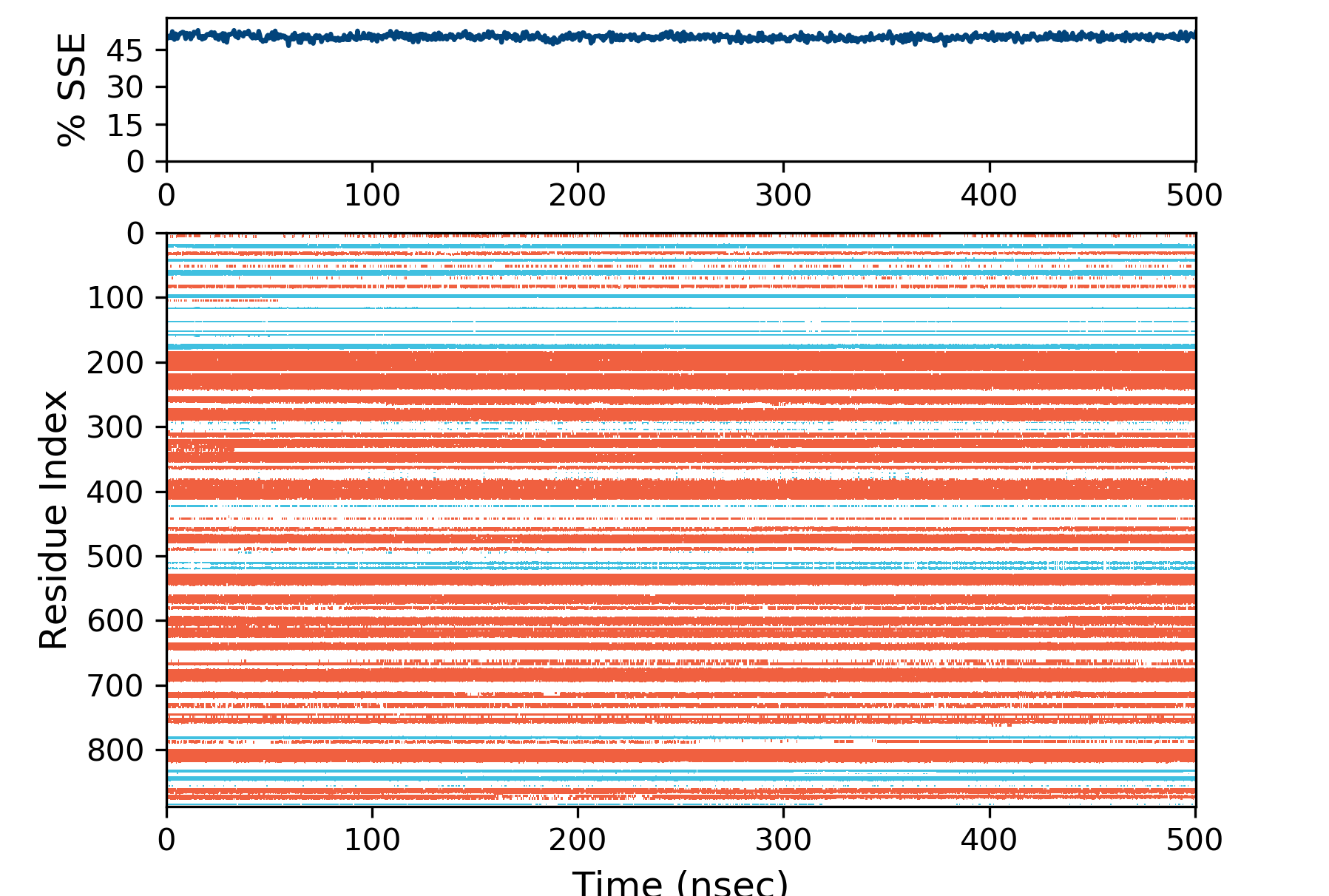


Figure SI 10: The SSE composition for each trajectory frame over the course of the simulation, and the plot at the bottom monitors each residue and its SSE assignment over time for CNT complex.


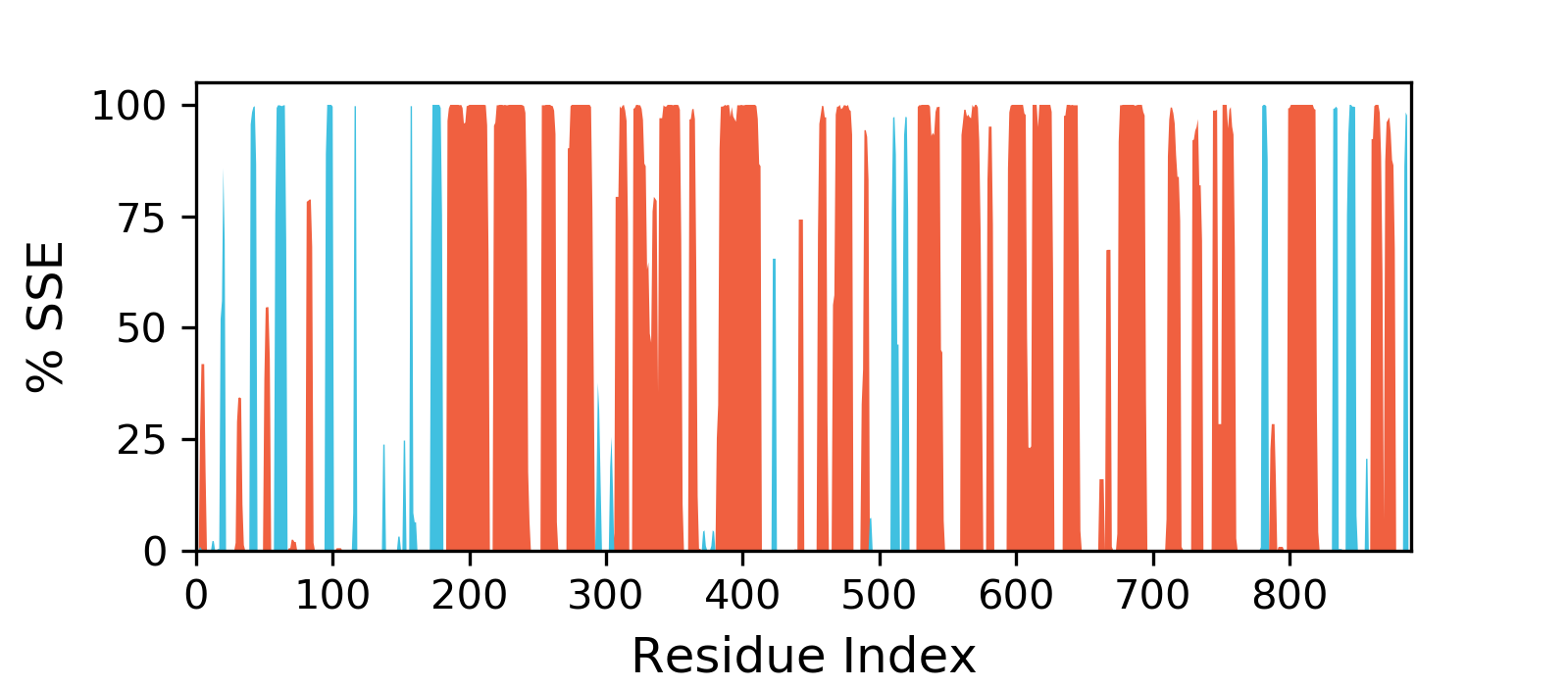


Figure SI 11: The SSE distribution by residue index throughout the protein structure for D693N


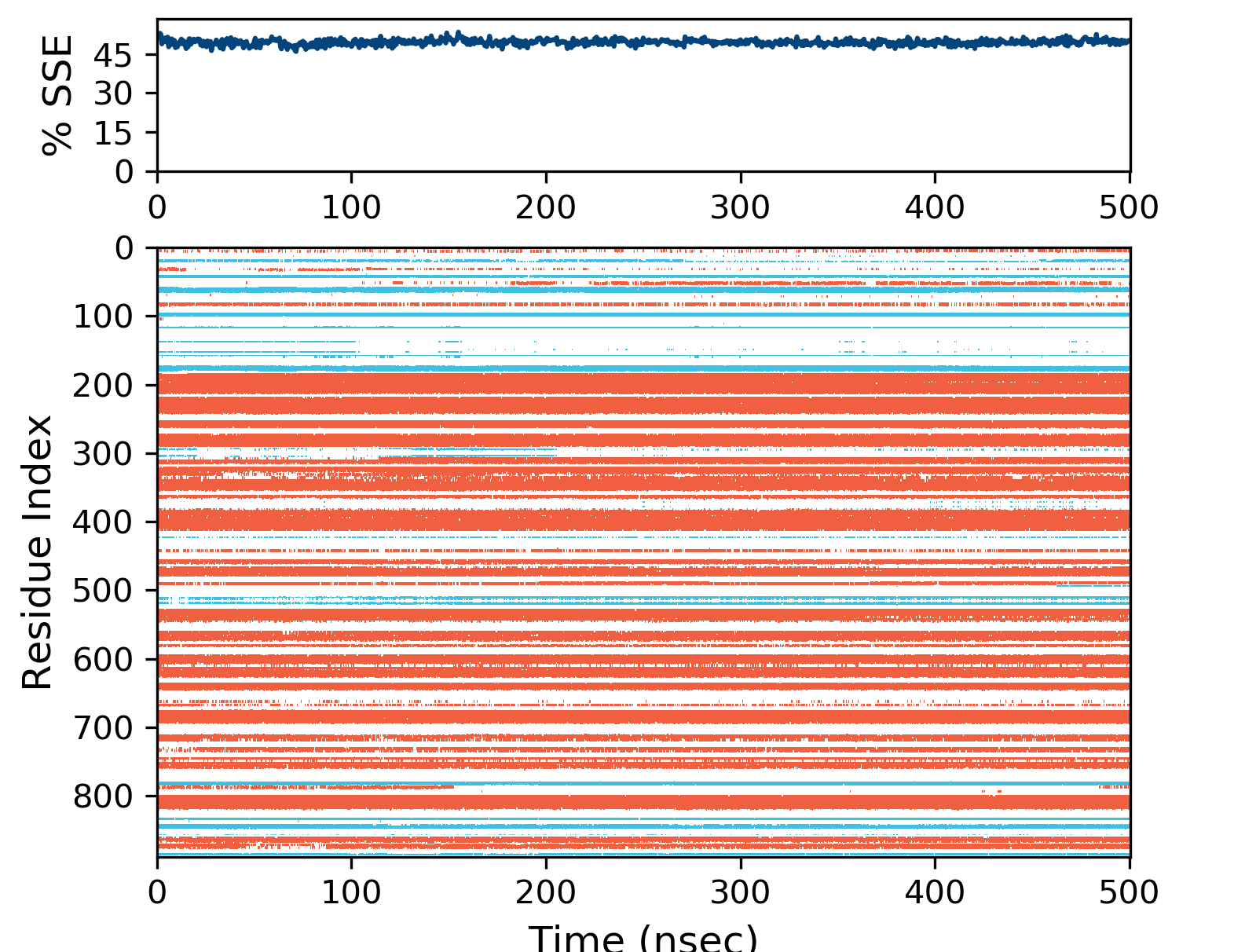


Figure SI 12: The SSE composition for each trajectory frame over the course of the simulation, and the plot at the bottom monitors each residue and its SSE assignment over time for D693N complex.


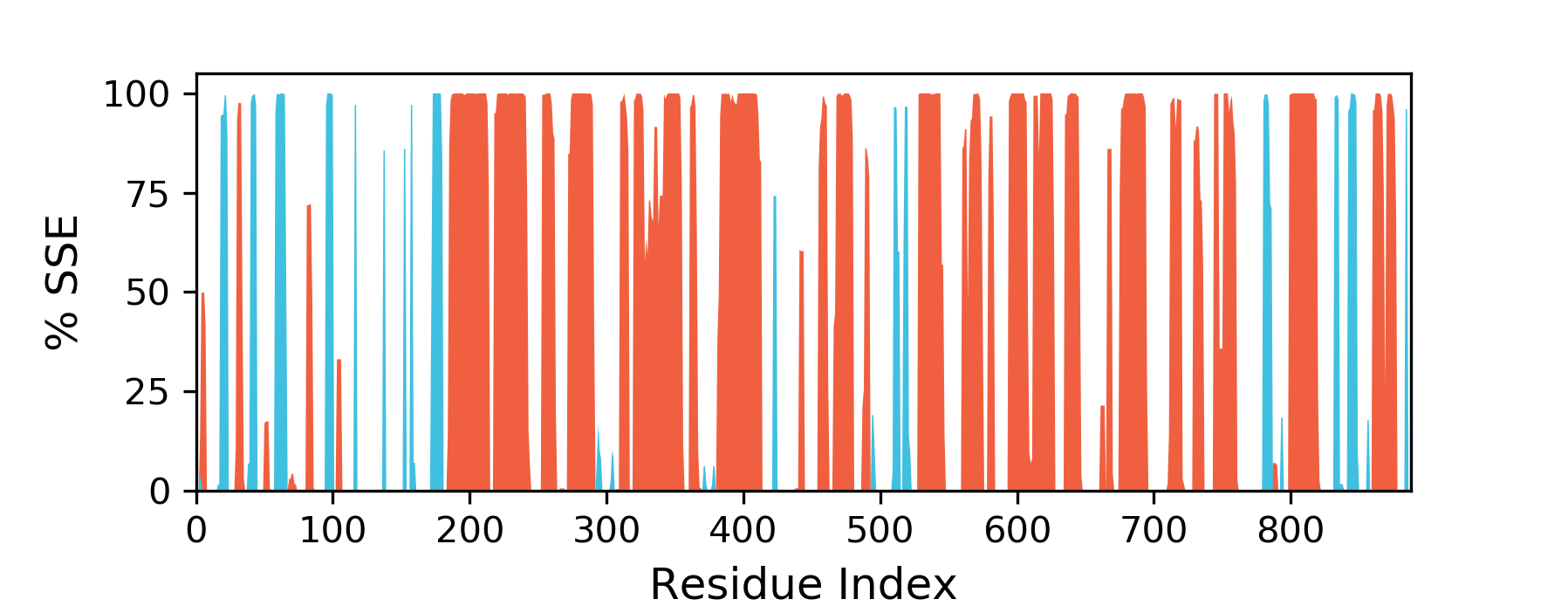


Figure SI 13: The SSE distribution by residue index throughout the protein structure for L320F


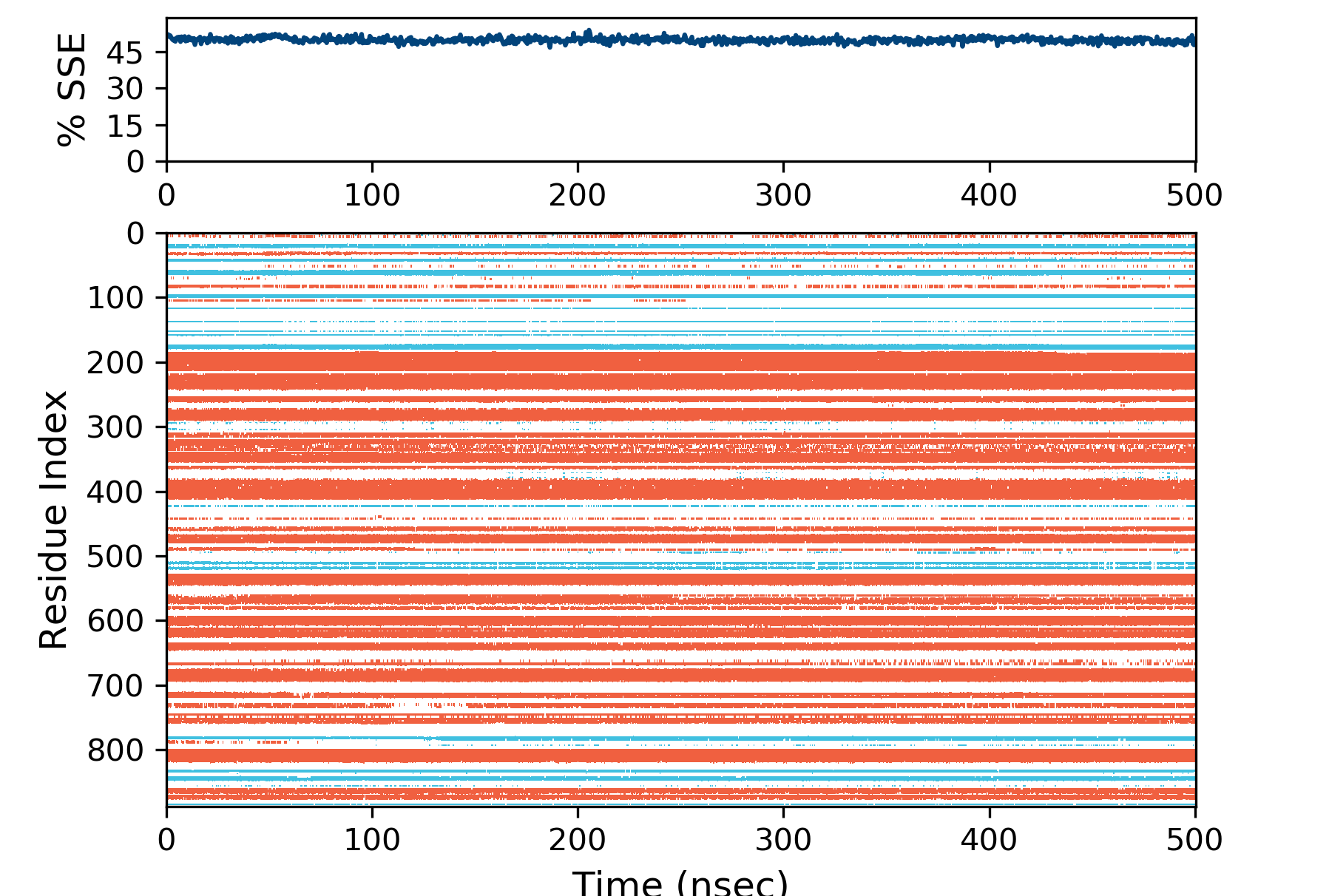


Figure SI 14: The SSE composition for each trajectory frame over the course of the simulation, and the plot at the bottom monitors each residue and its SSE assignment over time for L320F complex.


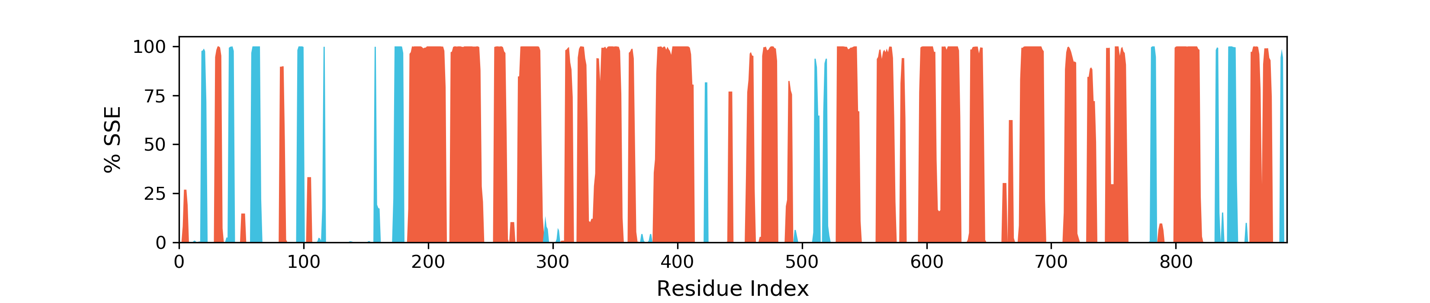


Figure SI 15: The SSE distribution by residue index throughout the protein structure for P84A


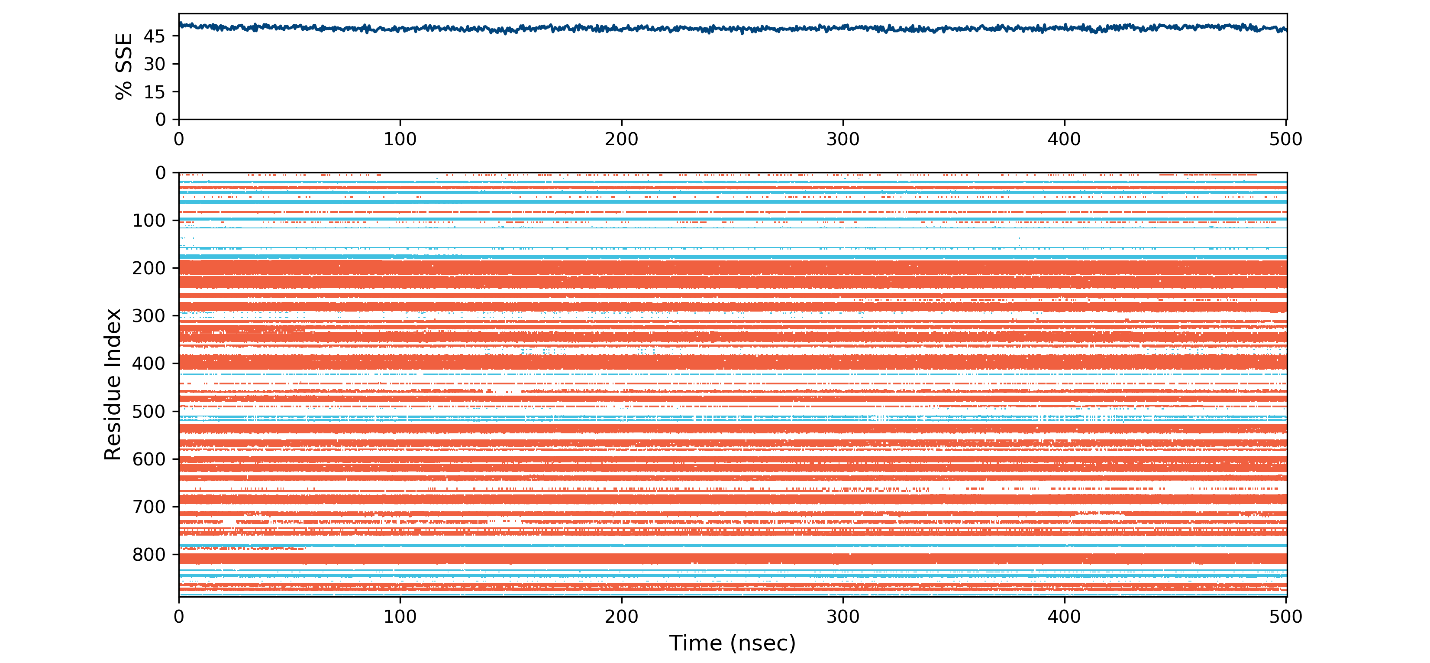


Figure SI 16: The SSE composition for each trajectory frame over the course of the simulation, and the plot at the bottom monitors each residue and its SSE assignment over time for P84A complex.


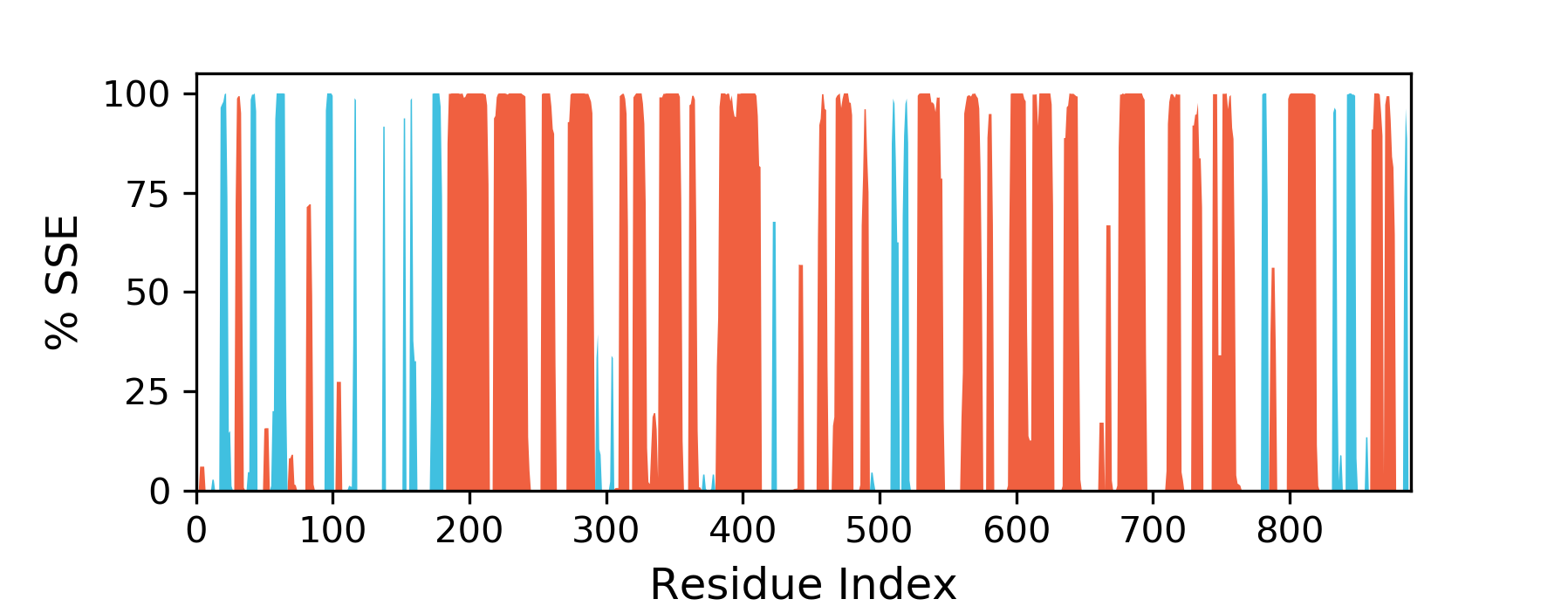


Figure SI 17: The SSE distribution by residue index throughout the protein structure for Q60E


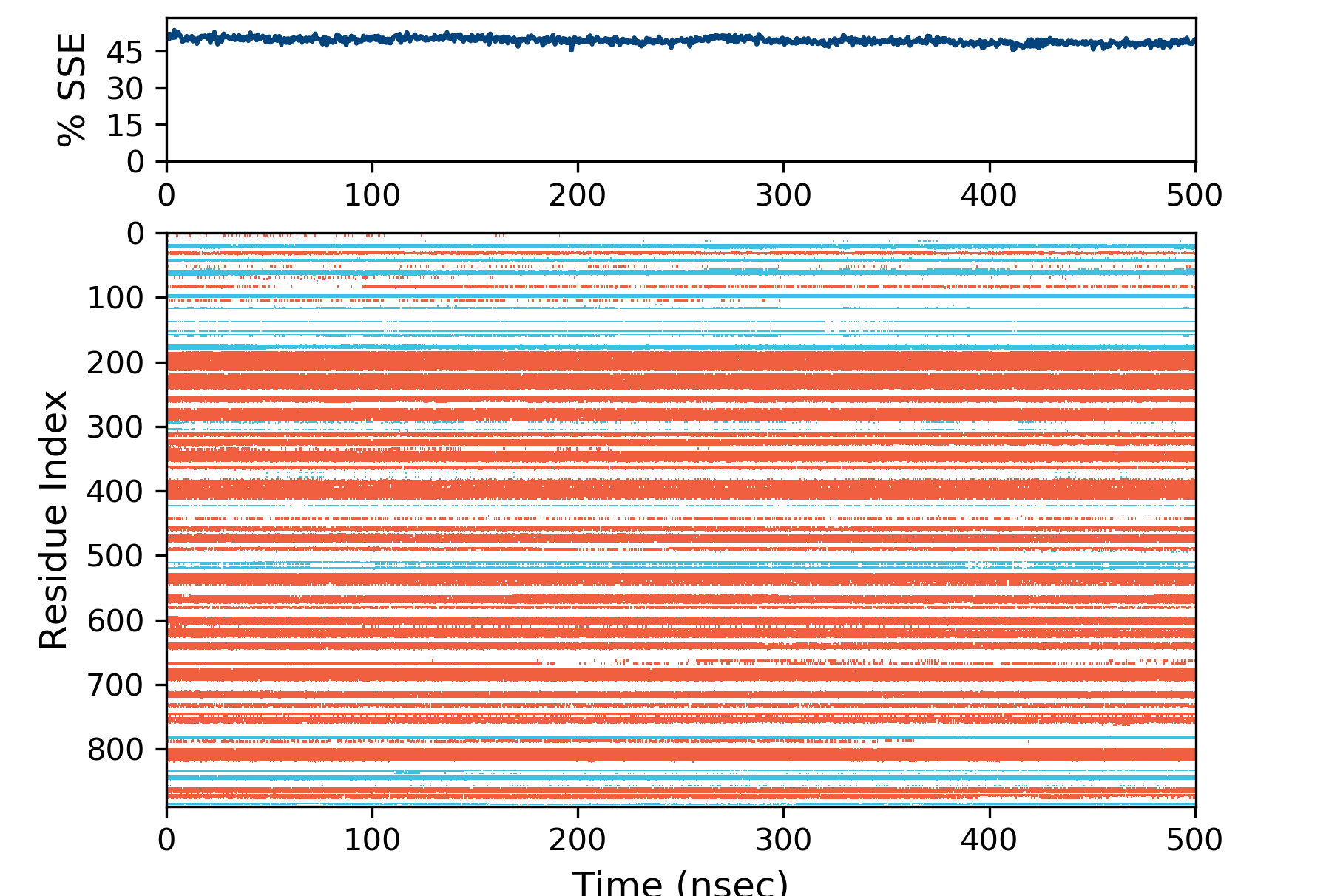


Figure SI 18: The SSE composition for each trajectory frame over the course of the simulation, and the plot at the bottom monitors each residue and its SSE assignment over time for Q60E complex.


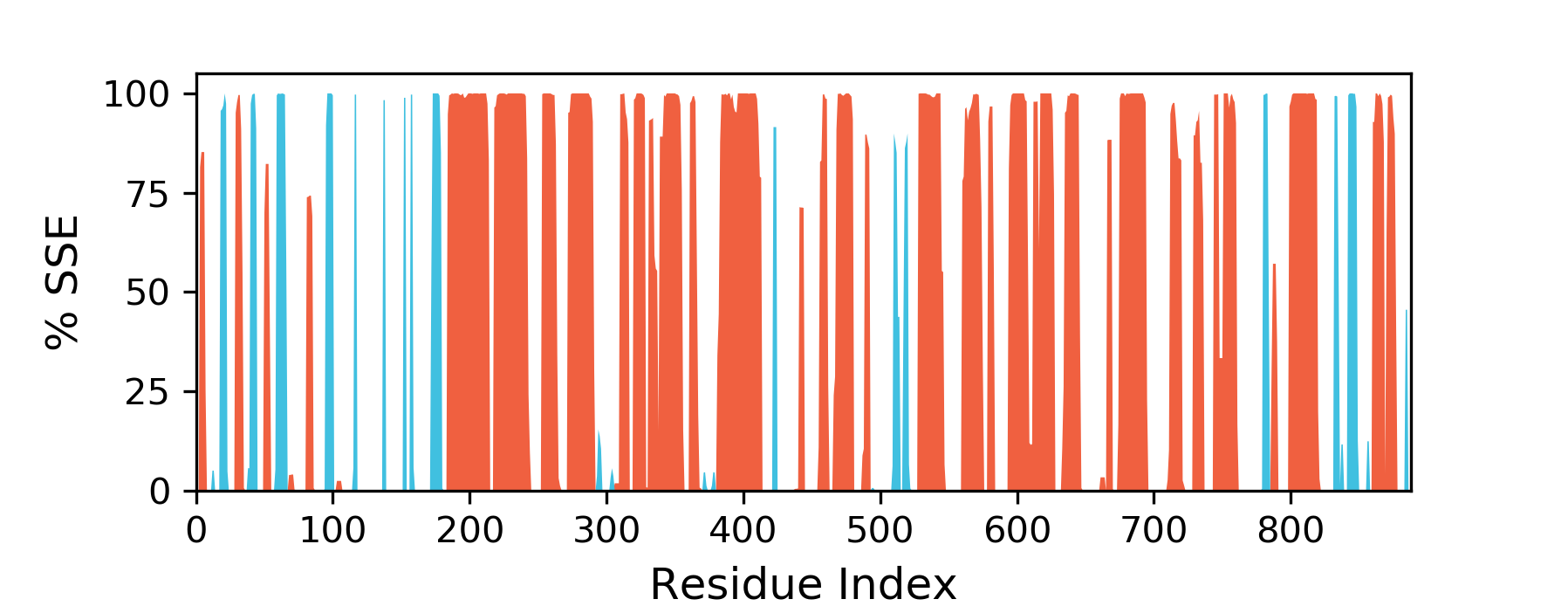


Figure SI 19: The SSE distribution by residue index throughout the protein structure for V491L


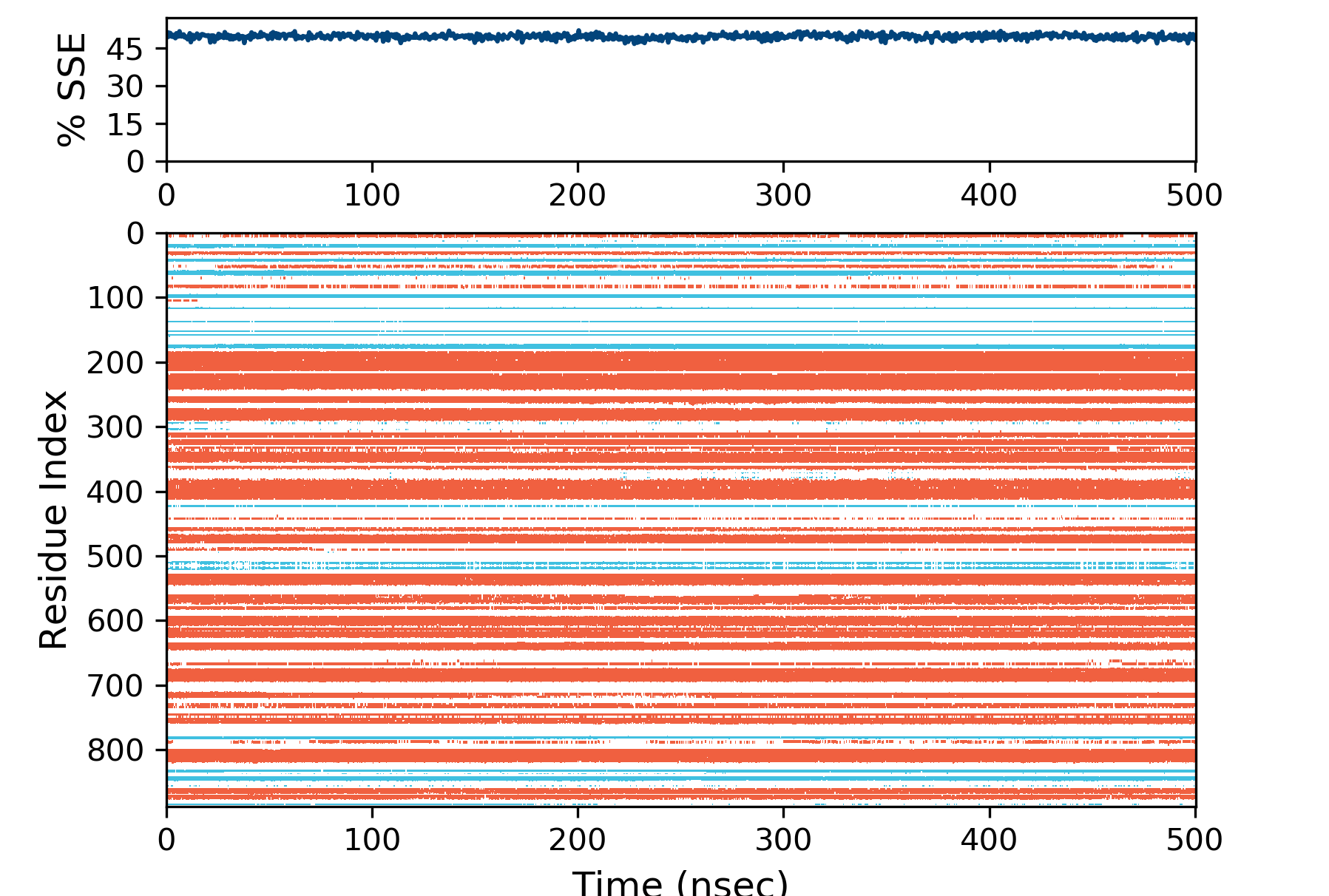


Figure SI 20: The SSE composition for each trajectory frame over the course of the simulation, and the plot at the bottom monitors each residue and its SSE assignment over time for V491L complex.

**References**

1. Release, S., 3: Desmond molecular dynamics system, DE Shaw research, New York, NY, 2017. *Maestro-Desmond Interoperability Tools, Schrödinger, New York, NY* **2017**.
2. Harder, E.; Damm, W.; Maple, J.; Wu, C.; Reboul, M.; Xiang, J. Y.; Wang, L.; Lupyan, D.; Dahlgren, M. K.; Knight, J. L.; Kaus, J. W.; Cerutti, D. S.; Krilov, G.; Jorgensen, W. L.; Abel, R.; Friesner, R. A., OPLS3: A Force Field Providing Broad Coverage of Drug-like Small Molecules and Proteins. *Journal of Chemical Theory and Computation* **2016,** *12* (1), 281-296.
3. Jorgensen, W. L.; Chandrasekhar, J.; Madura, J. D.; Impey, R. W.; Klein, M. L., Comparison of simple potential functions for simulating liquid water. *The Journal of Chemical Physics* **1983,** *79* (2), 926-935.
4. Neria, E.; Fischer, S.; Karplus, M., Simulation of activation free energies in molecular systems. *The Journal of Chemical Physics* **1996,** *105* (5), 1902-1921.
5. Release, S. d., 4. Desmond Molecular Dynamics System, DE Shaw Research. *Maestro-Desmond Interoperability Tools* **2016**.
6. Martyna, G. J.; Klein, M. L.; Tuckerman, M., Nosé–Hoover chains: The canonical ensemble via continuous dynamics. *The Journal of Chemical Physics* **1992,** *97* (4), 2635-2643.
7. Martyna, G. J.; Tobias, D. J.; Klein, M. L., Constant pressure molecular dynamics algorithms. *The Journal of Chemical Physics* **1994,** *101* (5), 4177-4189.
